# Metal ion-manipulated afterglow on rhodamine 6G derivative-doped room-temperature phosphorescent PVA films

**DOI:** 10.3389/fchem.2024.1441452

**Published:** 2024-09-12

**Authors:** Margarita Claudya Maida, Natsumi Sugawara, Airi Suzuki, Masato Ito, Yuji Kubo

**Affiliations:** Department of Applied Chemistry, Graduate School of Urban Environmental Sciences, Tokyo Metropolitan University, Hachioji, Japan

**Keywords:** room-temperature phosphorescence, tunable afterglow, Poly(vinyl alcohol), boronic acid, reversible metal-ligand coordination

## Abstract

The long-lived room-temperature phosphorescence (RTP) originating from thiophene boronate polyvinyl alcohol (PVA) has enabled the creation of metal-ion-responsive RTP films doped with spirolactam ring-containing rhodamine 6G (**1**). In this study, RTP-active PVA films, namely, **TDB@PVA** and **ATB@PVA**, were prepared through boronate esterification of thiophene-2,5-diboronic acid (**TDB**) and 5-acetylthiophene-2-boronic acid (**ATB**) with the diol units of PVA. The delayed emission properties were evaluated, revealing an emission band at 477 nm with a turquoise afterglow for **TDB@PVA** and at 510 nm with a green afterglow for **ATB@PVA** after UV light irradiation ceased. The photophysical properties were assessed using TD-DFT and DFT calculations at the B3LYP/cc-pVDZ level. *N*-(rhodamine-6G)lactam dye with a salicylimine unit (**1**) was doped into the RTP-based PVA films, producing a multicolored afterglow upon the addition of metal ions. This phenomenon is explained by a triplet-to-singlet Förster-type resonance energy transfer process from the cross-linked thiophene boronate in PVA to the metal-ion-activated colored form of **1**. This photophysical feature finds applicability in encryption techniques. Notably, the reversible metal-ligand coordination of **1** in the PVA system enabled a write/erase information process.

## 1 Introduction

Stimulus-responsive luminescent materials have garnered significant attention as smart materials, with their emission properties tunable by various external stimuli, including chemical species, temperature, water, pH, and mechanical force. (Gu and Ma, 2022; Lei et al., 2023). This responsiveness is closely linked to their sensing function, which has been widely investigated for fluorescence-based chemosensors used to efficiently detect chemically or biologically important analytes. (Wu et al., 2017; Deng et al., 2021; Sasaki et al., 2021; Krämer et al., 2022; Kumar et al., 2023). Recently, the demand for organic materials applicable to various state-of-the-art technologies has driven research into alternative emission modes for luminescent materials. (Zhang et al., 2018). Organic luminogens with room-temperature phosphorescence (RTP) offer the significant advantage of avoiding short-lived autofluorescence and scattering light caused by irradiation, facilitating practical applications (Li J. et al., 2022) such as time-gated bioimaging, (Zhao et al., 2015), information security, (Gmelch et al., 2019; Su et al., 2020), optoelectronics, (Kabe et al., 2016), among others. (Zhao et al., 2020; Shi et al., 2022). Unlike traditional fluorescence, triplet exciton-based emissive relaxation allows for long-lived delay emission, often visually detectable as an “afterglow” that persists after the excitation source is removed. (Yang et al., 2023). Such fascinating optical phenomena provide valid monitoring parameters. However, developing RTP materials with dynamic photophysical properties remains challenging. In principle, efficient RTP materials require both an increase in the triplet exciton population by facilitating intersystem crossing (ISC) between singlet and triplet states, as well as the suppression of nonradiative relaxation channels, including oxygen quenching. (Xu et al., 2023). Several approaches have been employed to create a rigid environment, including host-guest interactions, (Li et al., 2018; Yu et al., 2019; Wang et al., 2020), crystal forms, (Forni et al., 2018; Jia et al., 2020), H-aggregation, (An et al., 2015), intermolecular hydrogen bonding interactions, (Ma H. et al., 2019), and polymer matrices. (Gan et al., 2018). Although stimulus-triggered tuning in the afterglow has been investigated to date, including visual gas sensing, (DeRosa et al., 2017; Liu et al., 2022) temperature sensing, (Jin et al., 2020; Kawaguchi et al., 2024), metal ions detection, (Wei et al., 2020; Guo et al., 2022; Wu et al., 2022; Dai et al., 2023) volatile organic compounds detection, (Mei et al., 2022; Zhang et al., 2022), and NH_3_ and HCl detection, (Cheng et al., 2018) the environmental sensitivity of phosphorescence properties often hinders the dynamic manipulation of RTP materials.

**FIGURE 1:**
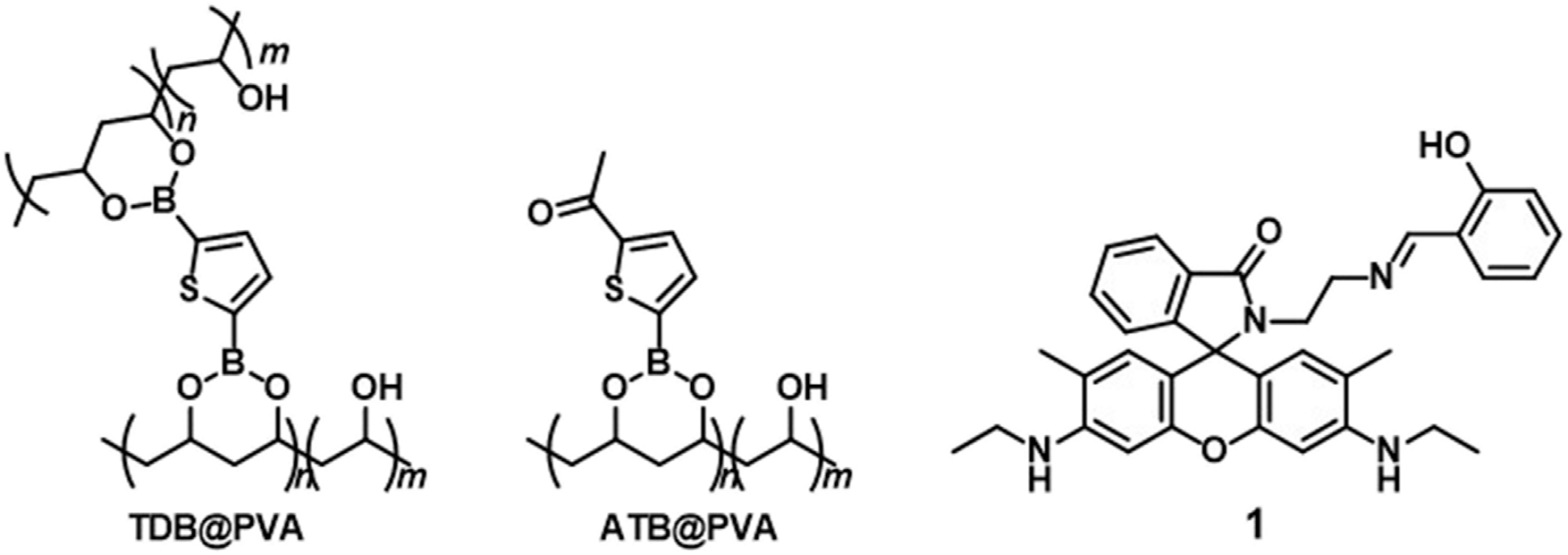
Chemical structures of **TDB@PVA**, **ATB@PVA**, and **1**.

Our interest in developing RTP-active materials motivated us to focus on boronic acid derivatives as phosphors. The empty p-orbitals of some boronic acids promote ISC and suppress the phosphorescence rate constant (*k*_P_), which results in outstanding long-lived RTP behaviors in rigid environments such as solid-state, (Chai et al., 2017; Kuno et al., 2017; Shoji et al., 2017; Yuasa and Kuno, 2018) doped films (Li D. et al., 2022) and doped crystals (Zhang et al., 2021; Zhou et al., 2021). Notably, the facile binding of boronic acids with the diol units of polyvinyl alcohol (PVA) is particularly advantageous for preparing RTP-active films. PVA is well-known for its mechanical flexibility and large-area production, making it suitable for a wide range of applications, such as food packaging, (Oun et al., 2022) biodegradable plastics, (Belay, 2023) and biocompatible materials in the medical and pharmaceutical field (Teodorescu et al., 2019). From the standpoint of RTP materials, PVA serves not only as a rigid matrix to protect triplet exciton but also contributes to accelerating intersystem crossing, (Liang et al., 2023) enabling the easy fabrication of RTP-based afterglow materials by suppressing vibrational dissipation and oxygen quenching of the excited triplet state (Al-Attar and Monkman, 2012; Kwon et al., 2014; Ma X. et al., 2019). Zhao et al. achieved ultralong single-molecule phosphorescence in a PVA polymer matrix and investigated the influence of aggregation, conformation, temperature, and moisture on monomer phosphorescence (Wu et al., 2020). Despite the superior properties of PVA matrices, the development of chemically stimulus-controllable RTP systems in PVA remains at the forefront. Previously, we prepared RTP-active thiophene boronate ester-cross-linked PVA (**TDB-PVA**) (refer to Figure 1), where the rigid environment of the PVA-based matrix stabilized the triplet state of the thiophene linker (Kanakubo et al., 2021). We thus hypothesized that doping *N*-(rhodamine-6G)lactam dye **1** (Lee et al., 2016) (Figure 1) into PVA would yield metal ion-responsive afterglow films (**1-TDB@PVA**). The metal ion-induced ring-opening reaction of **1** could induce the appearance of an absorption band with strong emission in the visible region, which would serve as an acceptor for a triplet-singlet Förster-type resonance energy transfer (TS-FRET) process (Sk et al., 2023) from the thiophene boronate in PVA, thus causing metal ion-manipulated multicolored afterglow. Furthermore, the reversible ring-opening/closing reaction of the spirolactam unit motivated us to investigate afterglow manipulation in our systems. With this in mind, pyrophosphate (PPi), which has a high binding affinity for metal ions, can dissociate the metal-ligand coordination. We postulated the dynamic manipulation of the RTP-based afterglow properties through reversible metal-ligand coordination using PPi, which has intriguing applications in the write/erase information process.

## 2 Experimental section

### 2.1 General

Nuclear magnetic resonance (NMR) spectra were measured on a JEOL JNM-ESC400 (^1^H: 400 MHz, ^11^B: 128 MHz). In ^1^H and ^11^B NMR measurements, chemical shifts (*δ*) are reported downfield from the internal standard Me_4_Si and external standard BF_3_∙OEt_2_, respectively. Mass spectrometry data were taken using a Bruker micrOTOF II-SDT1 spectrometer with atmospheric pressure chemical ionization (APCI) method. The absorption and emission were measured using Shimadzu UV-3600 UV/Vis/NIR spectroscopy and a JASCO FP-8500 spectrofluorometer, respectively. ATR-FTIR spectra were recorded on a JASCO FT/IR-4100 spectrometer with NaCl salt plate. The absolute photoluminescence quantum yields (*Φ*) for emission up to 650 nm were determined by JASCO FP-8500 spectrofluorometer equipped integral sphere (*ϕ* = 60 mm). Photographic images were taken with a digital camera (Canon, EOS Kiss X8i).

### 2.2 Materials

Unless otherwise indicated, reagents used for the synthesis were commercially available and were used as supplied. Thiophene-2,5-diboronic acid **TDB** and 5-acetylthiophene-2-boronic acid **ATB** were recrystallized with water. RTP-active PVA matrices **TDB@PVA** (Kanakubo et al., 2021) and 3′,6′-bis(ethylamino)-2-(2-(2-hydroxy-5-benzylideneamino)ethyl)-2′,7′-dimethylspiro[isoindolin-1,9′-xanthen]-3-one 1 (Lee et al., 2016) were prepared according to the method previously reported. The NMR data of **TDB@PVA** are as follows; ^1^H NMR (400 MHz, DMSO-*d*_6_, ppm) *δ* 1.23 – 1.52 (-CH(OH)-C*H*_2_-, and -CH(boronate ester)-C*H*_2_-), 3.82 – 3.88 (-CH(boronate ester), C*H*(OCOCH_3_) and -C*H*(OH)-), 4.24 – 4.25 (-O*H*), 4.49 (-O*H*), 4.89 (1H, -O*H*), 7.45 (thiophene-*H*); ^11^B NMR (128 MHz, DMSO-*d*_6_, ppm) *δ* 21.1.

### 2.3 Preparation of PVA film by doping 5-acetylthiophene-2-boronic acid (ATB@PVA)

An aqueous EtOH solution of PVA (0.25 unitM) and 5-acetylthiophene-2-boronic acid (0.1–2.0 mol%) was drop-casted on a silicon rubber plate and dried at room temperature overnight, and then dried *in vacuo*. The assignment was conducted by ATP-FTIR and ^1^H and ^11^B NMR measurements. ATR-FTIR spectra; 659 cm^‒1^ (boronate ester bond), ∼1270 cm^‒1^ (B‒O stretching), 1654 cm^‒1^ (C=O group). ^1^H NMR (400 MHz, DMSO-*d*_6_, ppm) *δ* 1.27 – 1.52 (-CH(OH)-C*H*_2_-, and -CH(boronate ester)-C*H*_2_-), 3.82–3.88 (-CH(boronate ester), C*H*(OCOCH_3_) and -C*H*(OH)-), 4.21 – 4.23 (-O*H*), 4.47 (-O*H*), 4.668 (-O*H*), 7.46 (thiophene-*H*), 7.89 (thiophene-*H*); ^11^B NMR (128 MHz, DMSO-*d*_6_, ppm) *δ* 15.6.

### 2.4 Fabrication of 1-TDB@PVA

A DMSO solution (500 μL) of PVA (number average molecular weight (M_n_) of 89,000–98,000; saponification number: ≥99%, 0.4 unitM. The concentration was based on the monomer unit.), thiophene-2,5-diboronic acid (2.0 mM), and **1** (48 μM) was drop-casted on micro slide glass with silicone rubber plate, heated at 60°C for 12 h, and dried *in vacuo*.

### 2.5 Evaluation of phosphorescence quantum yield

The delayed emission of RTP-active PVA films was measured by a JASCO FP-8500 spectrofluorometer equipped with an integral sphere (*ϕ* = 60 mm). The obtained spectra showed separated fluorescence and phosphorescence peaks. Then the integral emission intensities ranging from 400 nm to 650 nm were evaluated as phosphorescence quantum yield.

### 2.6 DFT/TD-DFT calculations.

Ground state and excited state geometries of thiophene-2,5-di(boronic acid)propane-1,3-diol diester (**TDB** ester) and 2-(4-acetylphenyl)-1,3,2-dioxaborinane (**ATB** ester) were optimized by density functional theory (DFT) at ωB97X-D3/def2-TZVP level with Orca 5.0 software, where spin-orbit coupling matrix elements (SOCMEs) and energy gaps (Δ*E*_ST_) between each singlet and triplet pair were computed. Natural transition orbital (NTO) calculation was conducted using B3LYP/cc-pVDZ level in the Gaussian 16 software. (Frisch et al., 2016). All geometries of the compounds at the ground state were fully optimized. Results of TD-DFT calculation are described in Supplementary Tables S1–S6. Optimized structures of ground state and excited states of **TDB** and **ATB** esters are indicated in Supplementary Tables S7–S12 as Cartesian coordinates.

## 3 Results and discussion

### 3.1 Preparation of RTP-active PVA films using thiophene boronic acids

Aqueous EtOH solutions of thiopheneboronic acids (**TDB** and **ATB**) and PVA (number average molecular weight (M_n_) of 89,000–98,000; saponification number: ≥99%, 0.4 unitM for **TDB** and 0.25 unitM for **ATB**. The concentration was based on the monomer unit.) were drop-casted and dried to produce **TDB@PVA** and **ATB@PVA**, respectively. Characterization was conducted using ATP-FTIR, revealing characteristic broad peaks ascribed to the boronate ester bond and B‒O stretching at approximately 650 – 660 cm^‒1^ and ∼1300 cm^‒1^, respectively. Additionally, a typical stretching band arising from the C=O group was observed at 1655 cm^‒1^ for **ATB-PVA**. Further information came from ^11^B NMR spectroscopy, where a broad signal at 15.6 ppm suggested that trigonal planar sp^2^ boron was adopted under the conditions. The average degrees of thiophene-based labeling are deduced to be 4.3 and 5.2 for **TDB@PVA** and **ATB@PVA**, respectively, based on the ^1^H NMR data (Supplementary Figure S1).

**FIGURE 2:**
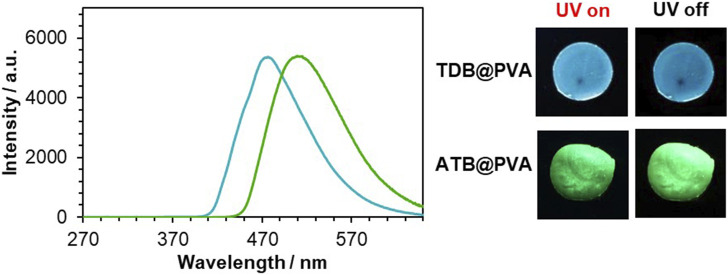
Delayed emission spectra of **TDB@PVA** (turqoise solid line) and **ATB@PVA** (green solid line). [**TDB**] = 0.2 mol%, [**ATB**] = 0.5 mol%. The afterglow images are also shown.

The delayed emission of the PVA films was recorded upon irradiation at 254 nm (Figure 2). **TDB@PVA** showed a significant RTP emission band at 477 nm when 0.2 mol% **TDB** was doped into PVA, and a turquoise afterglow was observed after ceasing the irradiation light. Based on our previous evaluation, the phosphorescence quantum yield (*Φ*_P_) is 6.3% with a phosphorescent lifetime (*τ*_P_) of 256 ms. (Kanakubo et al., 2021). On the other hand, the delayed emission of **ATB@PVA** was observed at 510 nm with *Φ*_P_ and *τ*_P_ values of 6.8% and 97.1 ms, respectively, upon excitation at 254 nm, when 0.5 mol% of **ATB** was grafted into PVA, as optimized conditions for RTP behavior (Supplementary Figure S2). Consequently, the color of the afterglow was green, and the emission band was red-shifted by 33 nm compared to that of **TDB@PVA**. Time dependency on the RTP properties was measured (Supplementary Figure S3). The intensity decreased as time passed; the decline ratios in the emission intensity were 7.7% and 16.6% for **TDB@PVA** and **ATB@PVA**, respectively, after 10 min passed. On the other hand, deaeration treatment for the films over 60 min enabled it to detect the emission recovery. Those results indicated that the RTP properties of the films are oxygen-sensitive. However, the thiophene-bridged PVA film is relatively stable, enabling it to acquire the photophysical data in this study.

**FIGURE 3:**
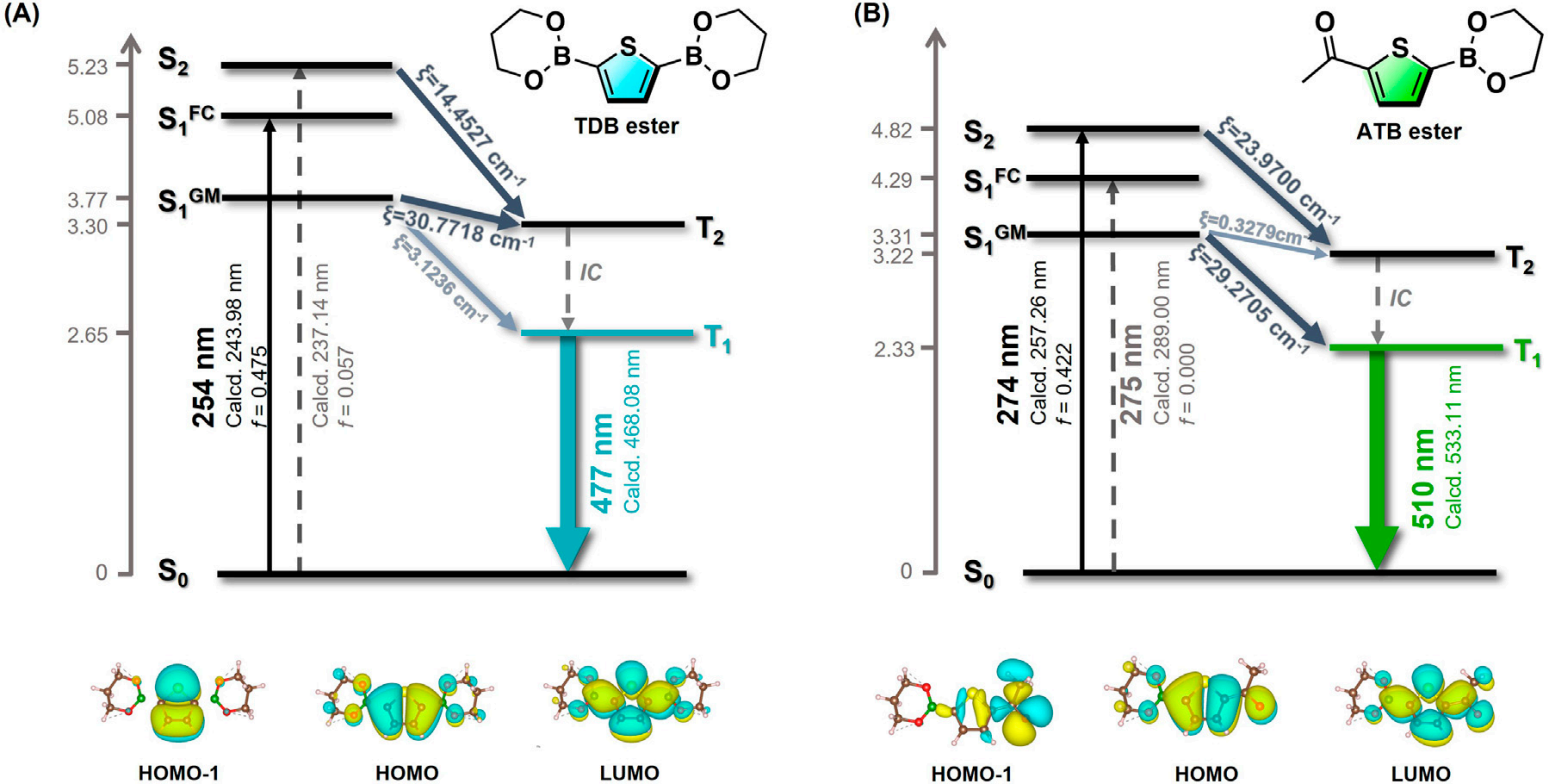
Jablonski diagrams and the related frontier molecular orbitals of **TDB** ester **(A)** and **ATB** ester **(B)**. The transition energies of S_1_^FC^ and S_1_^GM^ were evaluated based on the ground-state optimization and the S_1_ optimized structure, respectively. Transition energies of triplet states were assessed based on T_1_ optimized structure.

The impact of the acetyl substituent in **ATB@PVA** on the delayed emission was evaluated using TD-DFT and DFT calculations at the B3LYP/cc-pVDZ level. Given the limitations of our PC specifications, the current calculations for thiophene boronate PVA systems are based on an optimized single-molecule structure, where the effect of the polymer matrices is not considered. Thus, we calculated **TDB** ester and **ATB** ester as model compounds (Figure 3). The global minimized singlet energy level (S_1_^GM^) of **TDB** ester is calculated to be 3.77 eV, which is close to the T_2_ energy level. A small S_1_ ‒ T_2_ energy gap (0.47 eV) suggests a plausible ISC transition from S_1_ to T_2_. According to Kasha’s rule, the RTP property is postulated to be governed by the T_1_ → S_0_ transition, calculated at 468 nm (*λ*_calcd_), which is close to the experimental value. On the other hand, the S_0_ → S_1_ transition of **ATB** ester is a forbidden transition (HOMO‒1 → LUMO), being characterized by (n, π*) configuration, as inferred from NTO analysis. (Martin, 2003). Therefore, light excitation would prompt **ATB** ester to increase the population of the S_2_ state, followed by energy dissipation of S_2_ to S_1_ through internal conversion. An efficient ISC process to T_1_ would be possible based on El-Sayed’s rule, (El-Sayed, 1968), since T_1_ → S_0_ transition is characterized by (π, π*). This suggests that the carbonyl unit of the acetyl substituent contributes to an increase in the intersystem crossing rate. Although acetyl group of **ATB@PVA** could participate hydrogen bonding network to assist the rigidity of the system, the smaller lifetime of **ATB@PVA** (*τ*_P_ = 97.1 ms) allows us to consider that (n, π*) configuration of the acetyl group may govern the photophysical properties.

Given that RTP materials inherently exhibit a large Stokes shift in their photophysical properties, we note that **TDB@PVA** has an afterglow emission of less than 500 nm. (Zheng et al., 2023). In addition, given that a multicolor afterglow can be easily obtained by combining **TDB@PVA** with complementary emissive materials, we investigated the photophysical properties of metal ion-responsive RTP films using **TDB@PVA** doped with *N*-(rhodamine-6G)lactam dye **1**.

### 3.2 Metal ion-responsive rhodamine 6G-doped afterglow films

**FIGURE 4:**
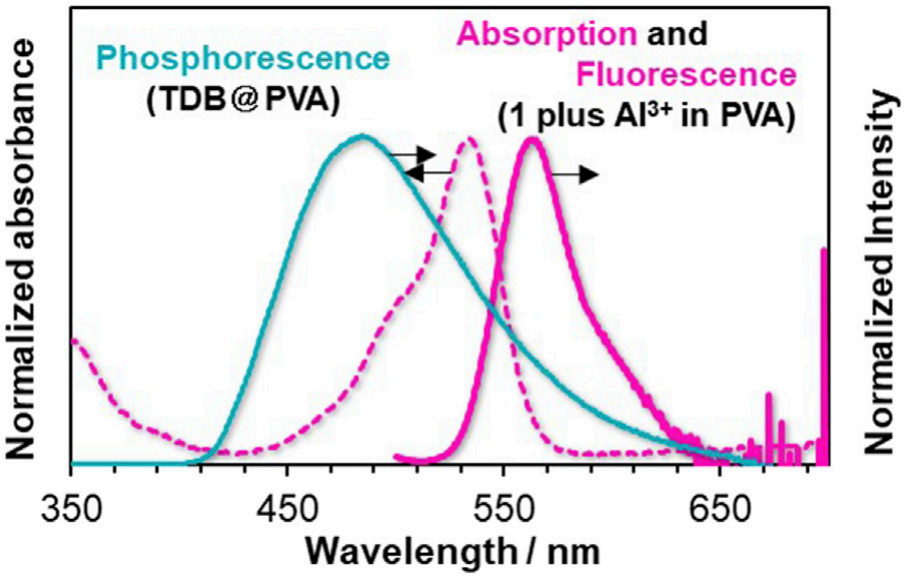
Phosphorescence spectra of **TDB@PVA** (turquoise solid line), and UV/Vis absorption (red dotted line) and emission spectra (red solid line) of Al^3+^-triggered colored **1** in PVA. The delay time is 50 ms.

We screened suitable rhodamine dyes to serve as metal ion-responsive TS-FRET acceptors in PVA. We note that *N*-(rhodamine-6G)lactam dye with salicylimine unit **1**; Supplementary Figure S4 shows the absorption and fluorescence spectra of **1** in MeOH/H_2_O (9:1 v/v), which showed a selective response toward metal ions (Supplementary Figure S5). As a preliminary assessment, the detection of Fe^3+^ was more responsive than Al^3+^ and Hg^2+^. Taking the absorption property of Fe^3+^ in the visible region into account (Supplementary Figure S6), our attention was focused on the Al^3+^-induced response to the photophysical properties. Adding Al^3+^ as a putative trivalent metal ion to the solution of **1** led to the appearance of an absorption band ranging from *ca*. 470–560 nm, whereas a fluorescence band was observed at 555 nm when excited at 530 nm. The association constant was determined by a non-linear curve fitting plot, giving 4.3 ×10^3^ M^–1^ (Supplementary Figure S7). However, it was found that Al^3+^-induced emission enhancement was almost inactive when **1** was doped in PVA, possibly due to an aggregation of **1** with the ring-opened structure in PVA (Supplementary Figure S8). Therefore, we tuned the internal microenvironment of PVA by modifying fabrication conditions. Subsequently, the corresponding RTP-active films prepared from a DMSO solution of **TDB** and **PVA**, instead of an EtOH/H_2_O solution, showed a much higher response as a factor of 4.2 than that of the film prepared using EtOH/H_2_O solution when 0.4 mM of Al^3+^ was added to each film (Supplementary Figure S8). To our delight, chemical stimuli-induced afterglow manipulation in PVA was conducted using **TDB@PVA** prepared from a DMSO solution of **TDB** and PVA (see the Experimental Section). The resultant film **TDB@PVA** has an RTP band at *ca*. 500 nm, which shows a negligible decrease in the emission intensity (≤21%) upon adding up to 0.6 mM of Al^3+^ (Supplementary Figure S9). It means that afterglow manipulation could be driven from reversible metal-ligand coordination on **1**. The photophysical interaction between the thiophene boronate linker and **1** in the PVA was also evaluated (Figure 4). The absorption band from the Al^3+^-triggered colored dye **1** overlapped with the delayed emission band of thiophene boronate. The photophysical properties could cause TS-FRET between them, possibly endowing the system with the dynamic function of a metal-ion-triggered change in the afterglow.

**FIGURE 5:**
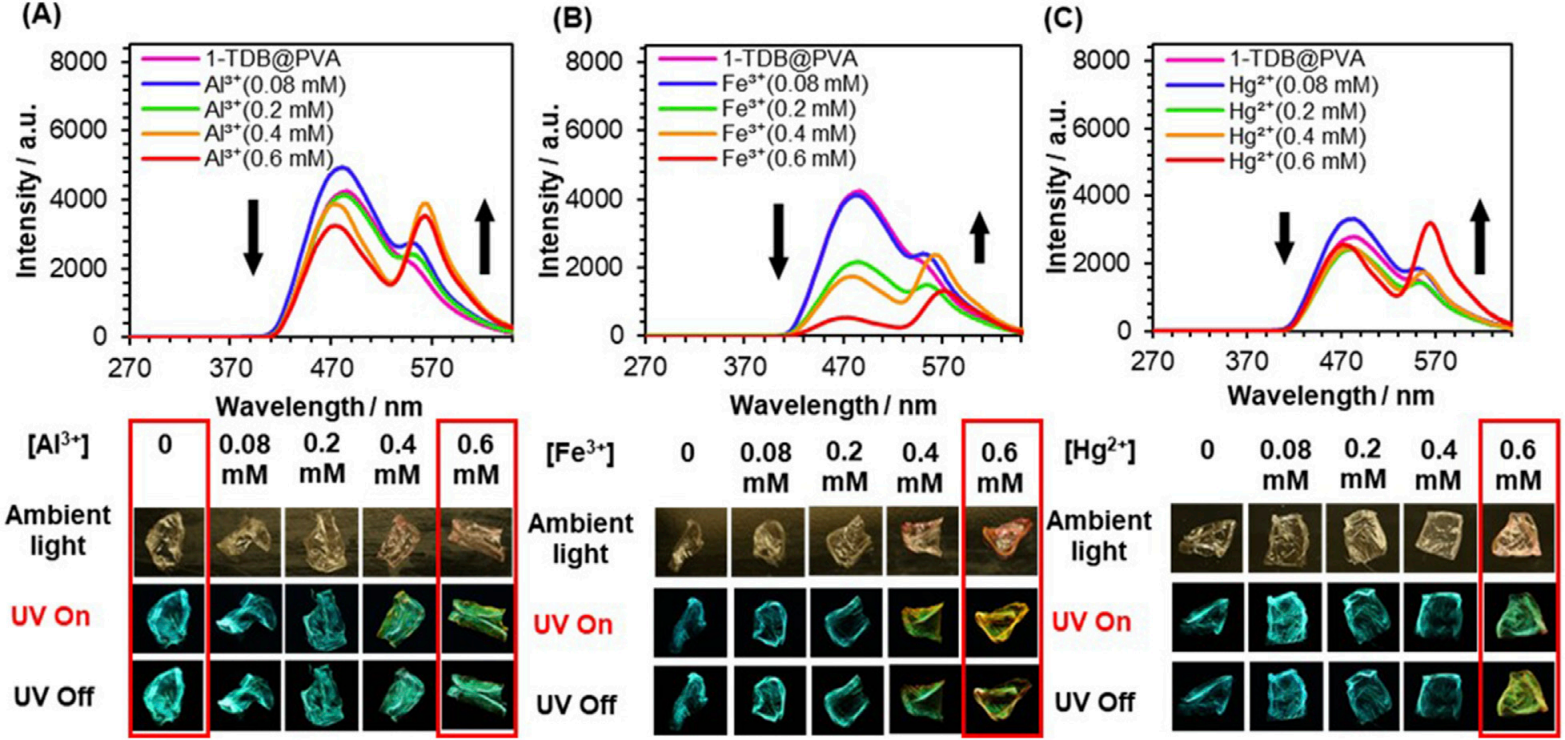
Delayed emission spectra of **1-TDB@PVA** upon adding Al^3+^ **(A)**, Fe^3+^ **(B)**, and Hg^2+^ **(C)**. [**TDB**] = 0.5 mol%, [M^n+^] = 0–0.6 mM. The images of the corresponding afterglow.

With the above in consideration, **1-TDB@PVA** was prepared by doping **1** (0.012 mol%) and **TDB** (0.5 mol%) with PVA. The stepwise addition of Al^3+^ to the film led to a change in the afterglow from turquoise to yellowish-green after ceasing UV irradiation at 254 nm. To understand this behavior, the delayed emission spectra of **1-TDB@PVA** were measured (Figure 5A). The emission intensity at 477 nm declined stepwise, whereas the intensity of the band at 565 nm significantly increased, accompanied by an isosbestic point at around 544 nm upon adding Al^3+^. The spirolactam ring-opening reaction of **1** was induced by Al^3+^, leading to an increase in the longer-wavelength emission at 565 nm through TS-FRET from the thiophene-linker unit of PVA to spirolactum ring-opened **1**. The presence of the FRET process was supported by a change in the phosphorescence lifetime of the system. When 0.6 mM of Al^3+^ was added into the PVA film, energy transfer efficiency was estimated to be 10.6% based on *E* = 1 ‒ *τ*_FRET_/*τ*_D_, (Hoshi et al., 2020), where *τ*_FRET_ and *τ*_D_ are the lifetimes of the donor–acceptor conjugate (**1-TDB@PVA** with Al^3+^) and donor (**1-TDB@PVA**), respectively (Supplementary Figure S10). The lifetime (*τ*) of compound **1** in **1-TDB@PVA** with 0.6 mM of Al^3+^ was measured to be 157.5 ms when irradiated at 254 nm. Although the value is slightly lower than that (*τ* = 179.5 ms) at 477 nm, the lifetime of the longer-wavelength region may be governed by that of the FRET donor. Utilizing the metal ion-dependent ring-opening reaction of **1** doped in the PVA system, delayed multicolored emission was achieved; the addition of Hg^2+^ and Fe^3+^ to the films changed the afterglow to yellowish green. In this context, a lower-intensity phosphorescence band was observed upon the addition of Fe^3+^, which was responsible for extreme quenching by Fe^3+^. This is presumably because Fe^3+^ absorbs the emission from thiophene boronate (Supplementary Figure S6). The relatively low response for Hg^2+^ (Figure 5C) can be interpreted by a low affinity of **1** with Hg^2+^, the association constant being 6.0 × 10^2^ cm^–1^ M^–1^ in MeOH/H_2_O (9:1 v/v) (Supplementary Figure S7). In this way, different color afterglow emissions were fairly observed by adding metal ions (0.6 mM) after ceasing the UV light; green for Al^3+^, orange for Fe^3+^, and yellowish green for Hg^2+^ (Figure 5).

**FIGURE 6:**
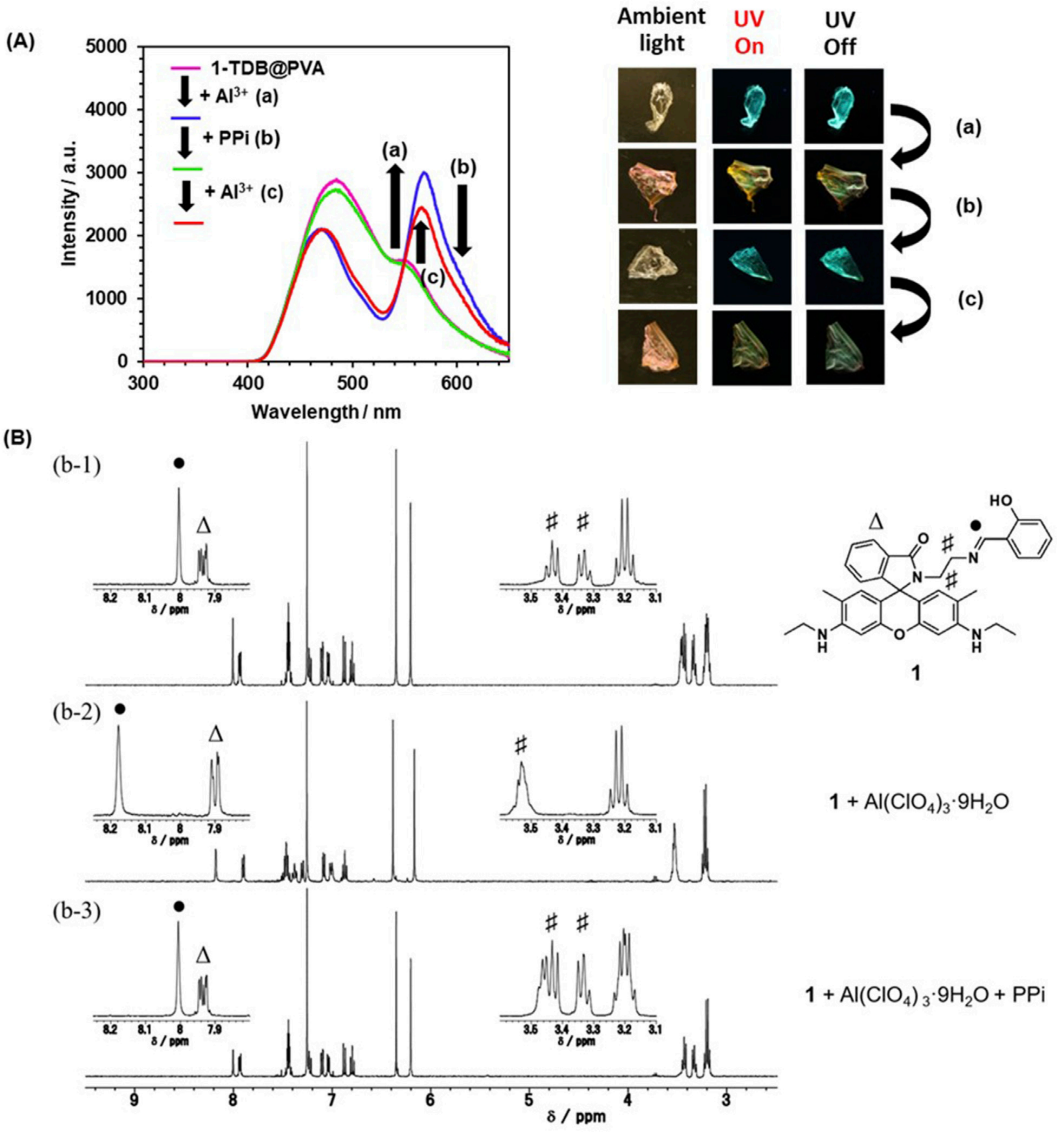
**(A)** Delayed emission spectra of **1-TDB@PVA** plus Al^3+^ (0.6 mM) before and after drop-casting PPi: Adding Al^3+^ (0.6 mM) to **1-TDB@PVA** path (a), PPi (0.3 mM) was drop-casted to **1-TDB@PVA** with Al^3+^ (path (b)) and adding Al^3+^ (0.6 mM) as the second run (path (c)). **(B)** ^1^H NMR spectra of **1-TDB@PVA** (b-1), **1-TDB@PVA** plus 4 equiv. of Al^3+^ (b-2), and an excess amount of PPi was added to (b-2) solution, then shaken in CDCl_3_ at room temperature (b-3).

Next, a reversible change in the afterglow was attempted through the dynamic metal-ligand coordination properties of **1**. Goswami et al. developed a naked-eye detection method for PPi by dynamic metal-ligand coordination with a rhodamine dye chelated with Al^3+^. (Goswami et al., 2013). In our case, an Al^3+^-induced yellowish-green afterglow film was drop-casted by PPi aqueous solution. After drying, the emission spectrum was superimposed on that of **1-TDB@PVA** (Figure 6A). And then the addition of Al^3+^ to the resultant film in the second run led to the appearance of an emission band at 565 nm. This reversible phenomenon is supported by a model experiment using NMR spectroscopy (Figure 6B). The proton resonance of benzylidene amino unit “●” was shifted significantly downfield by 0.172 ppm by adding Al(ClO_4_)_3_ in CDCl_3_, accompanying a slight shift (0.031 ppm) of one set of doublet (7.94 ppm, *J* = 4.8 and 2.2 Hz) due to rhodamine unit “Δ.” Furthermore, the proton resonance of the ethylene linker unit “#” in the range of 3.31–3.48 ppm was somewhat broadened. Taken together, these results strongly support the Al^3+^-triggered ring-opening reaction and coordination of **1**. In contrast, adding PPi to a solution of **1** plus Al^3+^ led to an almost complete recovery of these chemical shifts. The strong binding affinity between Al^3+^ and PPi can release metal ions from **1** to causing a ring-closure reaction. This indicates that PPi, as a chemical stimulus, controls the reversible metal-ligand coordination on **1**.

**FIGURE 7:**
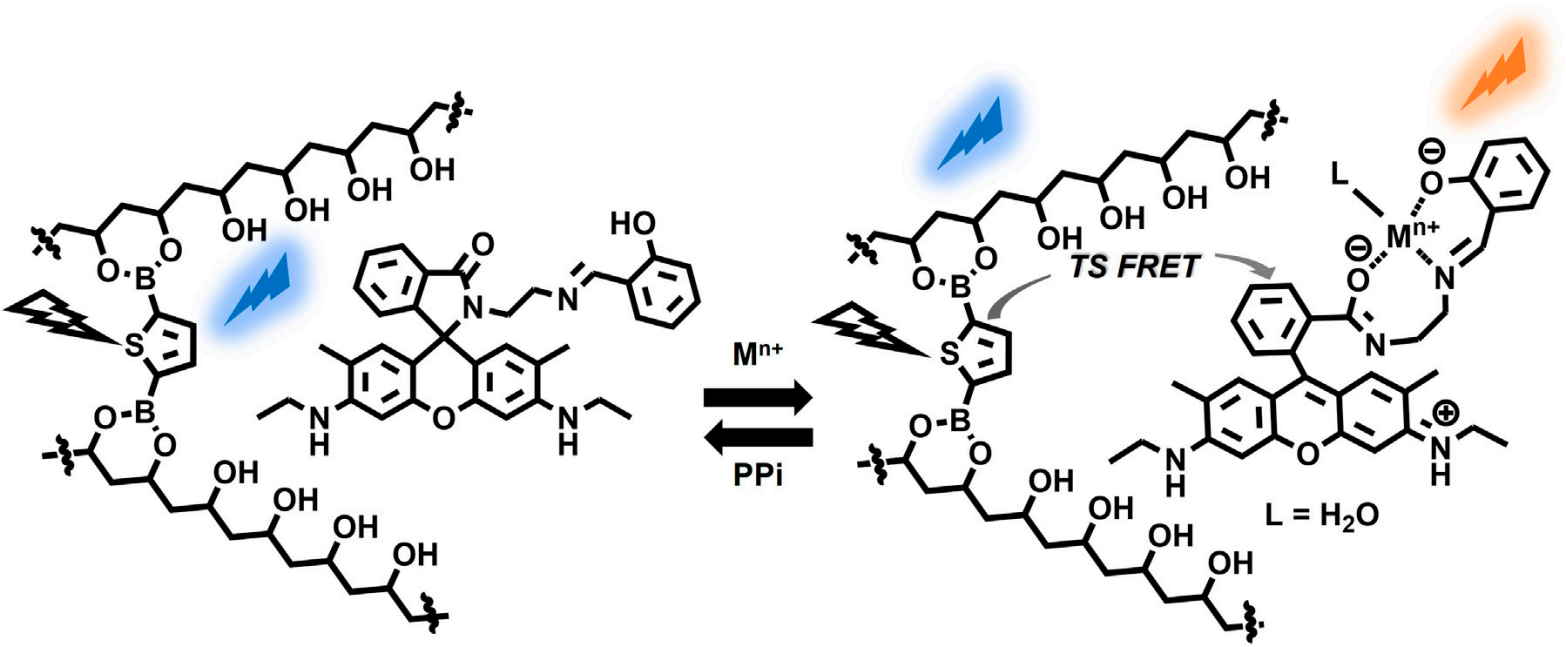
Plausible mechanism of chemical stimuli-induced afterglow manipulation with **1-TDB@PVA**.

A plausible working mechanism is illustrated in Figure 7. A metal ion-triggered spirolactam ring-opening reaction on **1** in PVA caused a TS-FRET process from the crosslinked thiophene boronate to the emission state of **1**, causing a change in the afterglow of the film. Reversible metal ion coordination on **1** enabled the manipulation of the afterglow properties through the dissociation of the metal ions by anions with a high affinity for **1**. This mechanism is highly applicable in encryption systems (Vide Infra).

### 3.3 Applications

**FIGURE 8:**
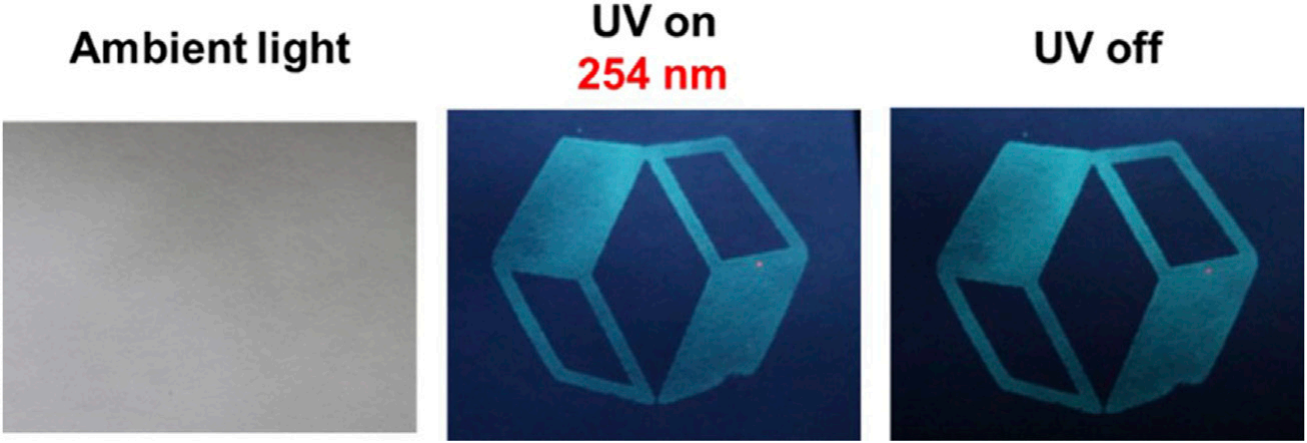
Images of the logo printed on filter paper under ambient light, UV irradiation, and after ceasing the UV light.

We prepared encrypted papers using **1-TDB@PVA** to investigate if **1-TDB@PVA** would serve as economical safety ink. As shown in Figure 8, the logotype was placed on a filter paper coated with DMSO ink composed of **1-TDB@PVA** using a silk screen printing technique. Although this information was not noticeable under ambient light, it was observed under UV irradiation at 254 nm (UV on), and after removal of the excitation (UV off).

**FIGURE 9:**
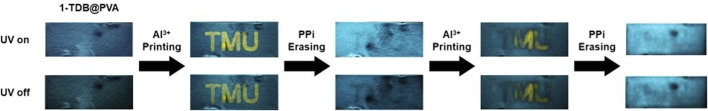
Schematic diagram of encryption systems with **1-TDB@PVA**. The information was invisible under ambient light. The letters “TMU” written by Al^3+^ aqueous solution were visible both under UV light irradiation and after ceasing UV light. The written information was erased after immersing the paper in an aqueous solution of PPi. The printing/erasing process was repeated.

The dynamic metal-ligand coordination of **1** in PVA motivated us to apply **1-TDB@PVA** to an information encryption system. Initially, **1-TDB@PVA** was coated on the filter paper, and the letters “TMU” were written on the paper with a DMSO solution of Al(ClO_4_)_3_ and then dried (Figure 9). Upon ceasing the UV light, the letters appeared as a yellow afterglow. The information was then erased by immersing the paper in an aqueous PPi solution and performing a desiccative process, although the **1-TDB@PVA**-based turquoise afterglow remained. This can be explained by the dissociation of Al^3+^ from dye **1** in PVA upon the addition of PPi, as inferred from a model experiment using ^1^H NMR (Figure 6B, Vide Supra). However, re-printing by adding Al^3+^ resulted in unclarity, which has made us consider the following reasons: (1) Immersing **1-TDB@PVA**-coated film in PPi aqueous solution could cause leaching of **1** doped; (2) Residual PPi on the filter paper may serve as a scavenger for Al^3+^ in the re-printable process. Although some improvement is desired to solve this issue, afterglow systems with reversible metal ion-ligand coordination would provide a potent approach for developing information encryption techniques.

## 4 Conclusion

Our ongoing program for developing chemical stimulus-tunable RTP films has led to the investigation of thiophene boronate-cross-linked PVA. The afterglow properties were influenced by the substituent groups on the thiophene skeleton, as evaluated by TD-DFT and DFT calculations. **TDB@PVA** exhibits RTP characteristics with a turquoise afterglow. Combined with *N*-(rhodamine-6G)lactam dye **1** that serves as a metal ion-responsive color element, color-tunable afterglow emissions were obtained through TS-FRET between thiophene boranate with a turquoise emission and the metal-ion-activated colored form of **1** in PVA. More impressively, the reversible metal-ligand coordination on **1** enabled afterglow manipulation in PVA by combining Al^3+^ and PPi as chemical stimuli. This approach was applied to information encryption films, allowing us to propose a write/erase system with **1-TDB@PVA** using dynamic coordination behavior.

## Data Availability

The data presented in the study are deposited in the PubCherm repository, accession number 126453.
