# Supplementary material for: Metal ion-manipulated afterglow on rhodamine 6G derivative-doped room-temperature phosphorescent PVA films
Source: Front Chem. 2024 Sep 12;12:1441452. doi: 10.3389/fchem.2024.1441452 (PMC11428105; doi:10.3389/fchem.2024.1441452)
Supplement: Supplementary file 1 [file DataSheet1.pdf]

Supplementary Material

Metal ion-manipulated afterglow on rhodamine 6G derivative-doped room-temperature phosphorescent PVA films

Margarita Claudya Maida, Natsumi Sugawara, Airi Suzuki, Masato Ito and Yuji Kubo*

Department of Applied Chemistry, Graduate School of Urban Environmental Sciences, Tokyo Metropolitan University, Hachioji, Tokyo, Japan

*** Correspondence:** yujik@tmu.ac.jp

1 General

NMR spectra were measured on a JEOL 400 MHz NMR spectrometer (^{1}H: 400 MHz, ^{13}C: 101 MHz, ^{11}B: 128 MHz). In ^{1}H, ^{13}C NMR, and ^{11}B NMR measurements, chemical shifts (δ) are reported downfield from the internal standard Me_4Si and external standard $BF_3 \cdot OEt_2$, respectively. Fast atom bombardment (FAB) mass spectrum was recorded on JEOL JMS-700 spectrometer and m-nitrobenzylalcohol was used as a matrix. High-resolution mass spectrometry (HRMS) analysis was performed using a Bruker MicrOTOF system with the APCI ionization method. The absorption and emission including time-gated spectra and lifetimes were measured using Shimadzu UV-3600 UV/Vis/NIR spectrophotometer and JASCO FP-8500 or JASCO FP-6500 spectrofluorometers, respectively. Quantum yields were measured by FP-8500 with an integrating sphere unit. Attenuated total reflectance (ATR) – IR spectrum was measured by a JASCO FT/IR-4600 type A spectrometer with ATR PRO 450-S attachment. Photographic images were taken with a digital camera (Canon EOS Kiss X8i).

2 Determination of the average degree of thiophene labeling in TDB@PVA and ATB@PVA

^{1}H NMR measurement of **TDB@PVA** with a higher concentration of **TDB** (1.0 mol%) was conducted to obtain a clear spectrum, where the thiophene protons at 7.45 ppm and the methylene protons (1.23 – 1.52 ppm) in the PVA backbone appeared in the ratio of 1:2, considering the thiophene-linked structure (Figure S1a). The number of **TDB** units per PVA of **TDB** (0.2 mol%)-containing PVA, as the average degree of labeling, can be estimated by the equation below:

$$\frac{I(TDB)}{I(PVA)} \times 2 \times DP \times 0.2 \quad \cdots\cdots \ (1)$$

where *I(TDB)* and *I(PVA)* denote the integration values of thiophene protons (1.0 mM) and methylene protons in the PVA, respectively. *DP* denotes degree of polymerization. Given that number average molecular weight of PVA (M_n = 89,000 – 98,000) and the molecular weight of the repeating unit of

PVA (M = 44), the average number of **TDB** units per PVA is deduced to be 4.3 for 0.2 mol% of **TDB**-containing PVA.

Similar procedure was applied to the determination of the number of **ATB** units per PVA in 0.5 mol% **ATB**-containing PVA using ^{1}H NMR spectrum of **ATB@PVA** (1.0 mol%) in DMSO-d_6. (Figure S1b), where the thiophene protons (7.46 and 7.89 ppm) and the methylene protons (1.27 – 1.52 ppm) on the PVA backbone appeared in the ratio of 1:1. Thus, the number of **ATB** units per PVA of **ATB** (0.5 mol%)-containing PVA, as the average degree of labeling, can be estimated by the equation below:

$$\frac{I(ATB)}{I(PVA)} \times DP \times 0.5 \quad \cdots\cdots \ (2)$$

where *I(ATB)* and *I(PVA)* denote the integration values of thiophene protons (1.0 mM) and methylene protons in the PVA, respectively. Subsequently, the average number of acetylthiophene unit in 0.5 mol% of **ATB** containing PVA was estimated to be 5.2.

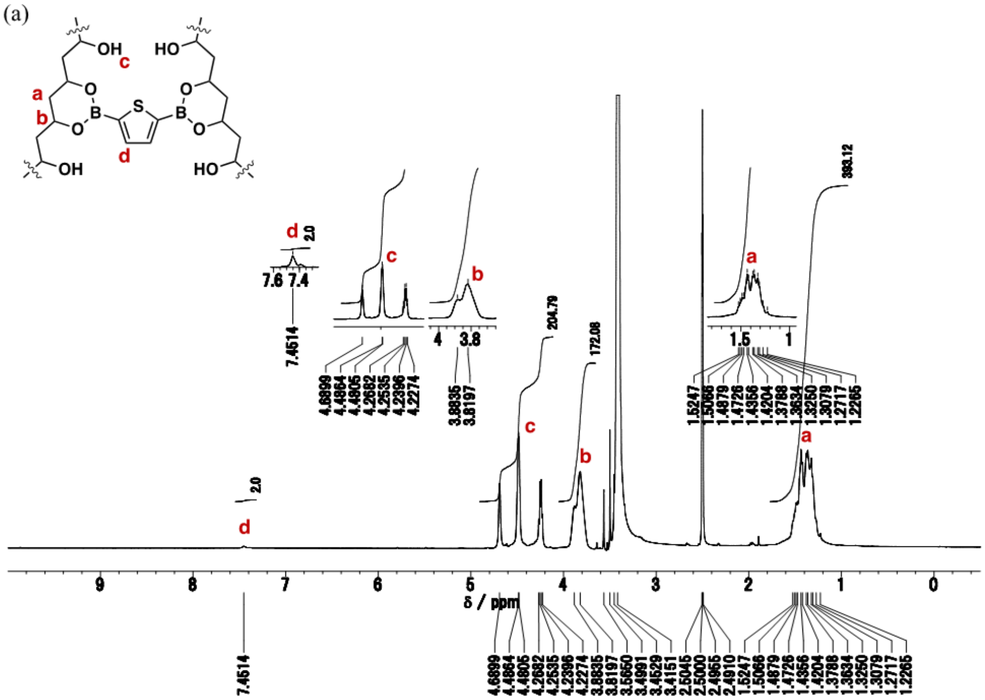

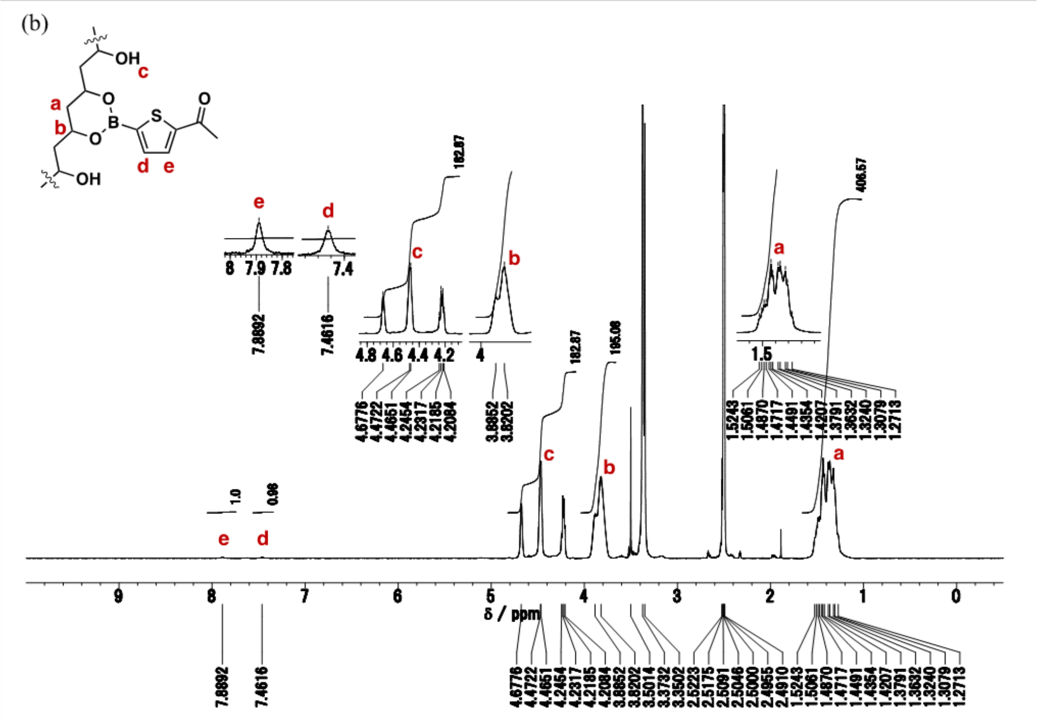

Figure S1. ^{1}H NMR spectra (400 MHz) of **TDB@PVA** (1.0 mol%) (a) and **ATB@PVA** (1.0 mol%) (b) in DMSO-d_6.

3 TD-DFT calculation

Ground state geometries of **TDB** and **ATB** esters were optimized by density functional theory (DFT) at ωB97X-D3/def2-TZVP level with Orca 5.0 software. These molecular structures and orbitals were visualized using Avogadro 1.2.0 software. Frequency calculations were also calculated for each optimized structure with same TD-DFT level and confirmed that there are no imaginary modes. Spin-orbit coupling matrix elements (SOCMEs) and energy gaps (ΔE_{ST}) between each singlet and triplet pair were also computed by TD-DFT at ωB97X-D3/def2-TZVP level with Orca 5.0 software. Excited state structures were optimized by time-dependent DFT at the same TD-DFT level. Frequency calculations were also calculated for each optimized structure with same TD-DFT level and confirmed that there are no imaginary modes. Natural transition orbital (NTO) was performed by Gaussian 16. Results of TD-DFT calculation are described in Table S1-S4. Optimized structures of the ground state and excited states of **TDB** and **ATB** esters are indicated in Table S5-S10 as Cartesian coordinates.

4 Synthesis of rhodamine-derivatives

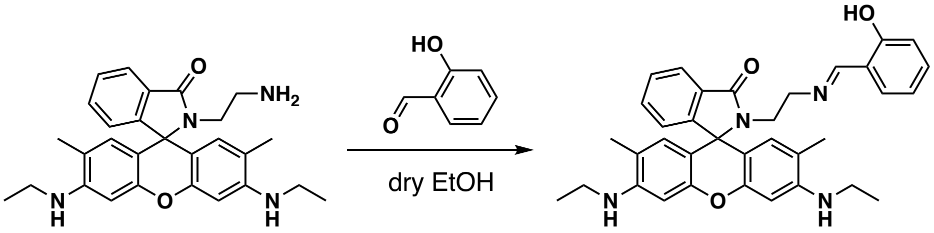

Scheme S1. Synthesis of rhodamine 6G-derived dyes.

2-(2-aminoethyl)-3',6'-bis(methylamino)spiro[isoindoline-1,9'-xanthen]-3-one (Compound 2)

To a solution of rhodamine 6G (1.21 g, 2.52 mmol) in EtOH (25 mL), ethylenediamine (3.75 mL, 56.2 mmol) was added dropwise while stirring. The mixture was heated at 80°C with stirring for 15 h. After cooling to room temperature, the resultant mixture was collected by filtration and dried *in vacuo*. Obtained pale pink powder was washed by EtOH to give the title compound as a pale pink crystal in 85% of yield. ^{1}H NMR (400 MHz, $CDCl_3$): δ (ppm) 7.94 – 7.90 (m, 1H), 7.48 – 7.42 (m, 2H), 7.07 – 7.03 (m, 1H), 6.34 (s, 1H), 6.22 (s, 2H), 3.50 (t, J = 5.1 Hz, 2H), 3.20 (qd, J = 7.2 Hz, J = 5.1 Hz, 4H), 3.15 (t, J = 6.8 Hz, 2H), 2.34 (t, J = 6.8 Hz, 2H), 1.90 (s, 6H), 1.33 (t, J = 7.2 Hz, 6H); FAB-MS: *m/z* = 457 $[M + H]^+$.

(*E*)-2-(2-((2-hydroxybenzylidene)amino)ethyl)-3',6'-bis(methylamino)spiro[isoindoline-1,9'-xanthen]-3-one (Compound 1)

A mixture of **2** (0.95 g, 2.10 mmol) and 2-hydroxybenzaldehyde (0.84 mL, 7.90 mmol) in dry EtOH (35 mL) was heated at 80°C with stirring for 7 h. The reaction mixture was concentrated under reduced pressure and dried *in vacuo*. Obtained orange powder was recrystallized by EtOH to give the title compound as an orange crystal in 42% of yield. ^{1}H NMR (400 MHz, $CDCl_3$): δ (ppm) 8.01 (s, 1H), 7.96 – 7.91 (m, 1H), 7.47 – 7.41 (m, 2H), 7.24 – 7.21 (m, 1H), 7.10 (dd, J = 7.6 Hz, J = 1.6 Hz, 1H), 7.06 – 7.01 (m, 1H), 6.88 (dd, J = 8.2 Hz, 0.6 Hz, 1H), 6.79 (ddd, J = 7.5 Hz, J = 7.4 Hz, J = 1.0 Hz, 1H), 6.35 (s, 2H), 6.20 (s, 2H), 3.47 – 3.41 (m, 4H), 3.33 (t, J = 7.7 Hz, 2H), 3.20 (qd, J = 7.0 Hz, J = 4.8 Hz, 4H), 1.84 (s, 6H), 1.32 (t, J = 7.1 Hz, 6H). ^{13}C NMR (101 MHz, $CDCl_3$): δ (ppm) 168.3, 165.7, 161.0, 153.6, 151.6, 147.4, 132.5, 131.9, 131.1, 131.0, 128.4, 128.0, 123.8, 122.8, 118.7, 118.2, 117.9, 116.9, 105.9, 96.5, 64.9, 57.1, 40.9, 38.3, 16.7, 14.7; HR APCI-MS: *m/z* = 561.2916 $[M + H]^+$.

5 Preparation of the PVA films

5.1. Prepared by EtOH/H_2O solution

An aqueous EtOH solution of PVA (0.25 unitM) and aryl dopant (*e.g.*, **TDB** and **ATB**) was drop-casted on a silicon rubber plate, dried at room temperature overnight, and then dried *in vacuo*.

5.2. Prepared by DMSO solution

The film was fabricated by drop-casting a DMSO solution (500 μL) of commercial PVA (number average molecular weight (M_n) of 89000 – 98000; saponification number = 99%, 0.40 unitM) and aryl dopant (*e.g.*, **TDB** and **ATB**) 0.5 mol% (2.0 mM) then heating (60ºC, overnight) and drying *in vacuo* (2h).

6 Concentration-dependent time-gated PL spectra of ATB@PVA

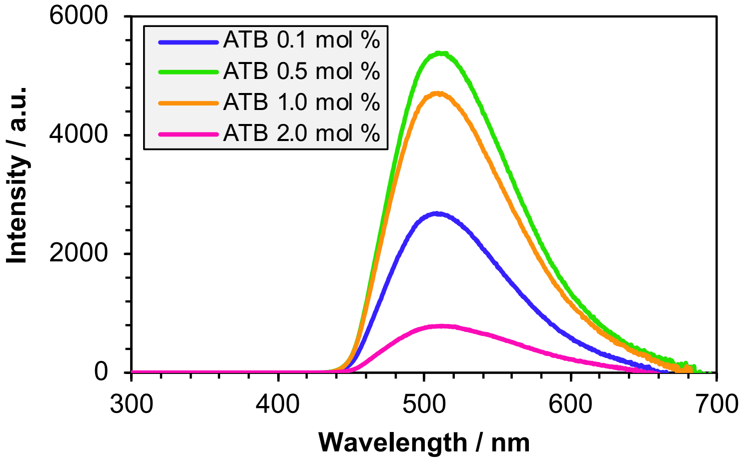

Figure S2. Concentration-dependent time-gated PL spectra of **ATB** in PVA film without normalization (λ_{ex} = 254 nm, delay time: 50 ms, room temperature).

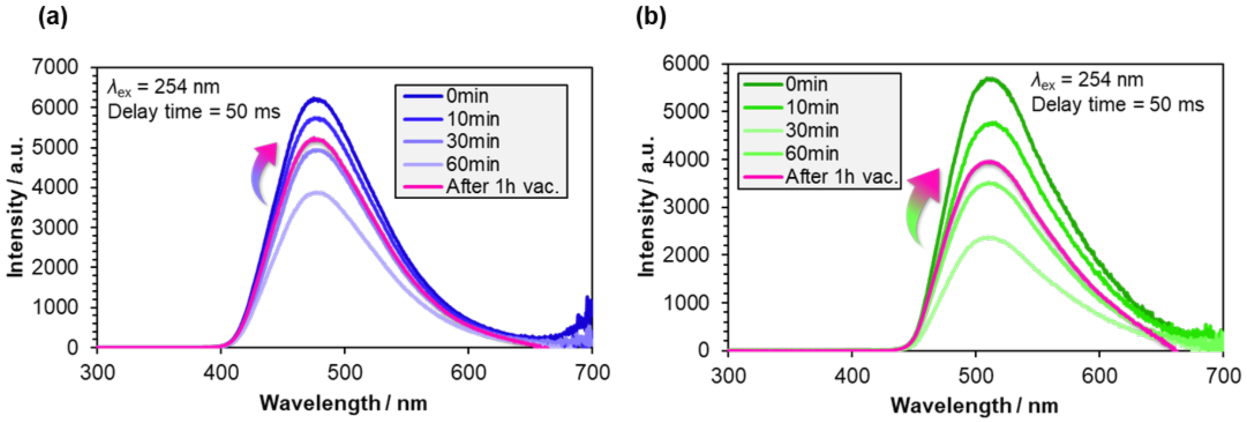

Figure S3. Time-dependency in the delayed emission spectra of **TDB@PVA** (a) and **ATB@PVA** (b). [**TDB**] = 0.2 mol%. [**ATB**] = 0.5 mol%. λ_{em} = 254 nm. Delayed time = 50 ms.

7 UV/Vis absorption and fluorescence spectra of 1 upon addition of Metal ions in MeOH/H_2O

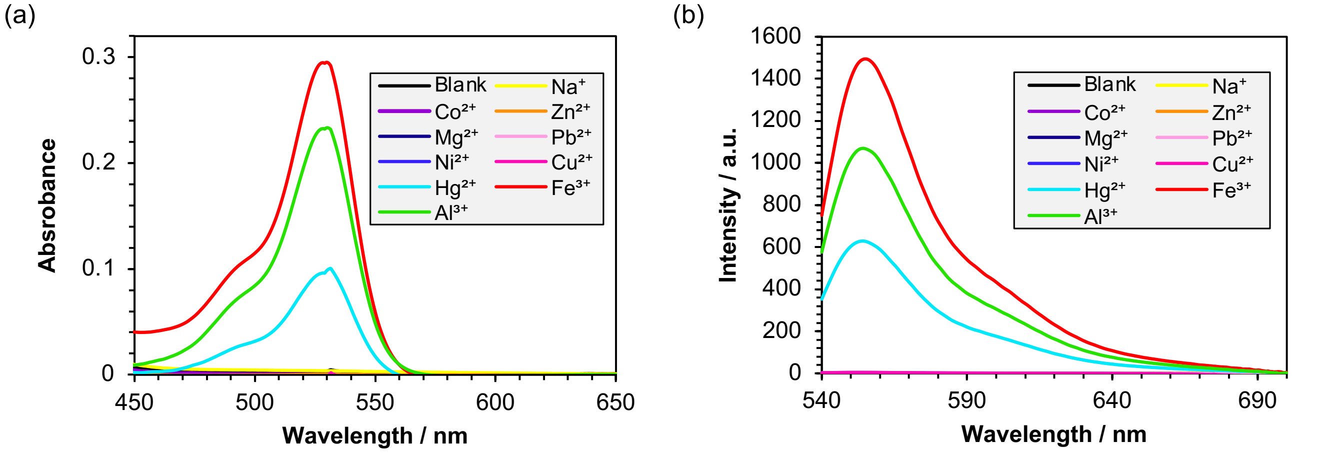

Figure S4. UV/Vis absorption (a) and steady-state PL spectra (b) of **1** upon addition of 5 equiv. of several metal ions as perchlorates salts in MeOH/H_2O (9:1 v/v). (λ_{ex} = 530 nm, [**1**] = 50 μM, room temperature)

8 Image of the corresponding photographs of 1 upon addition of metal ions in MeOH/H_2O

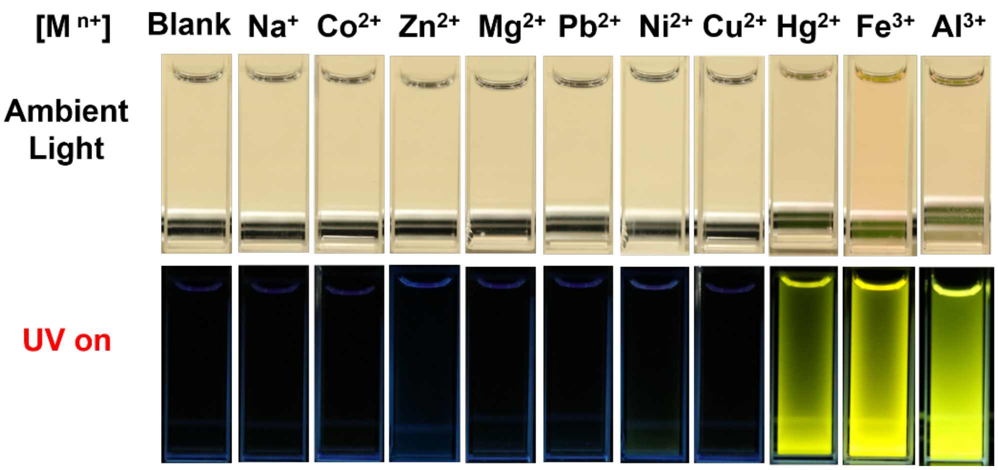

Figure S5. Image of the corresponding photographs of **1** upon addition of several metal ions in MeOH/H_2O (9:1 v/v) (λ_{ex} = 254 nm, [**1**] = 50 μM, room temperature).

9 UV/Vis absorption spectra of $Fe(ClO_4)_3$ in MeOH/H_2O

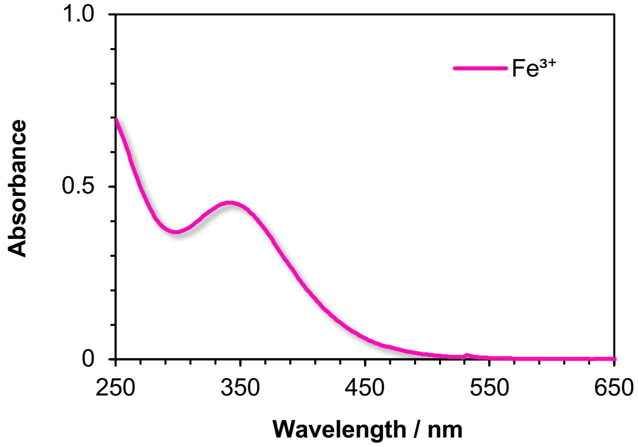

Figure S6. UV/Vis absorption spectra of $Fe(ClO_4)_3$ in MeOH/H_2O (9:1 v/v). ([$\mathbf{Fe(ClO_4)_3}$] = 250 µM, room temperature)

10 Titration of 1 with $Al(ClO_4)_3$ or $Hg(ClO_4)_2$ in MeOH/H_2O

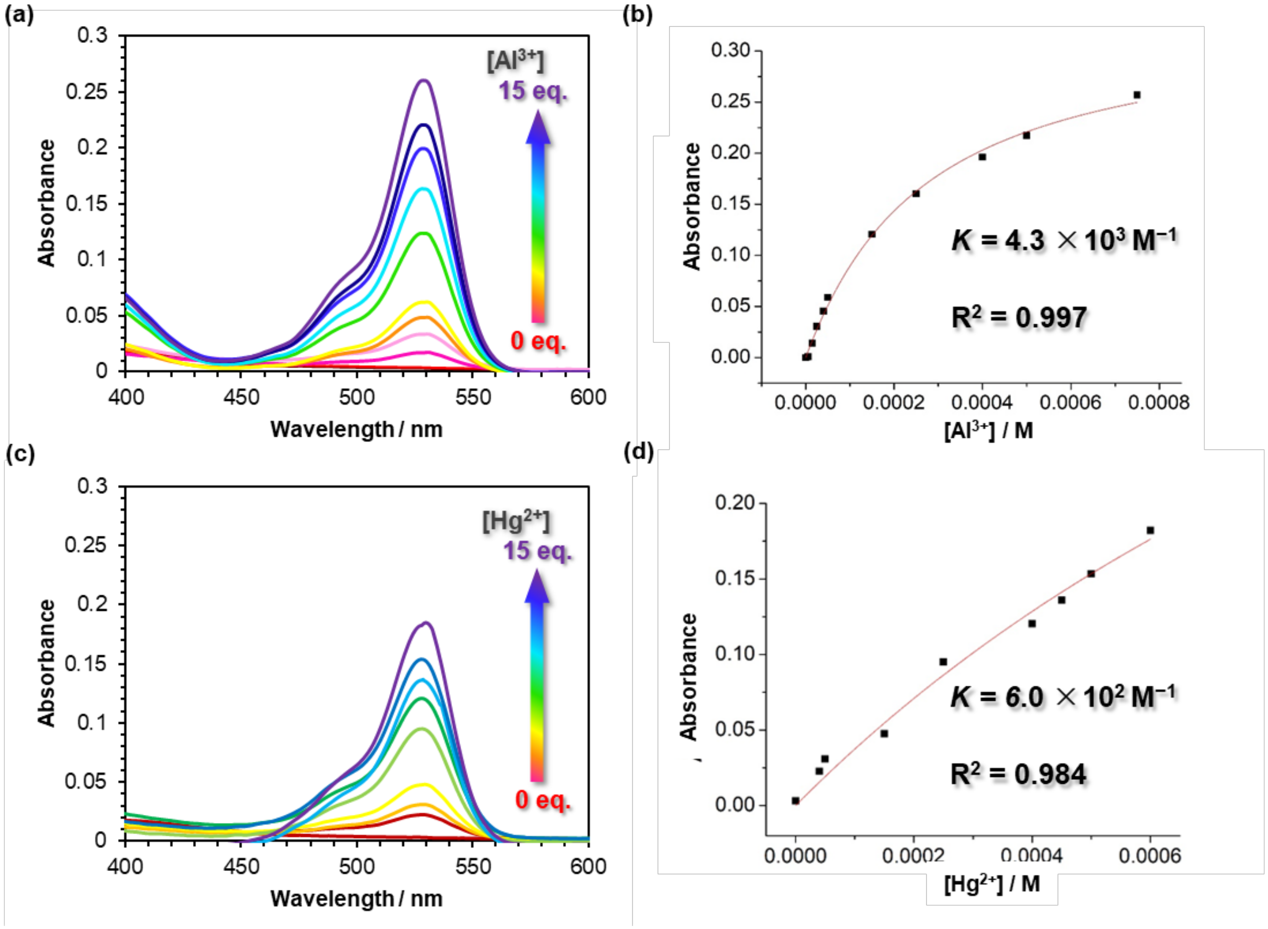

Figure S7. Concentration-dependent UV/Vis absorption spectra of **1** with $Al(ClO_4)_3$ (a) or $Hg(ClO_4)_2$ (c) in MeOH/H_2O (9:1 v/v) and UV/Vis titrations of **1** with incremental amounts of $Al(ClO_4)_3$ (b) or $Hg(ClO_4)_2$ (d) in MeOH/H_2O (9:1 v/v). The association constants were determined based on absorption spectra at 528 nm ([**1**] = 50 μM, room temperature).

11 Concentration dependency of 1 in PVA

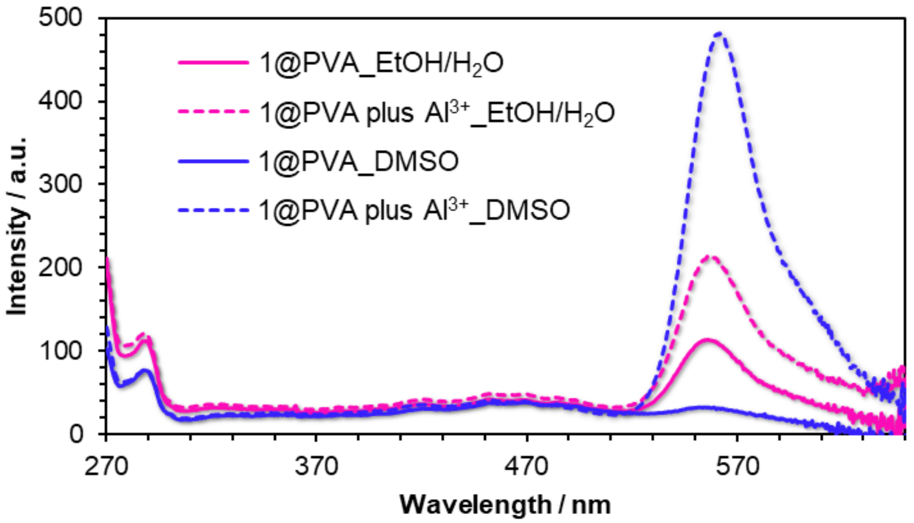

Figure S8. Steady-state spectra of **1@PVA** before and after the addition of Al^{3+}: **1@PVA** prepared from EtOH/H_2O solution before Al^{3+} addition (pink solid line) and after Al^{3+} addition (pink dotted line). **1@PVA** prepared from DMSO solution before Al^{3+} addition (blue solid line) and after Al^{3+} addition (blue dotted line). [**1**] = 48 μM (0.012 mol%). [Al^{3+}] = 0.4 mM as perchlorate salts in aqueous solution (λ_{ex} = 254 nm, room temperature).

12 Change in the PL and time-gated spectra of TDB@PVA by adding Al^{3+}

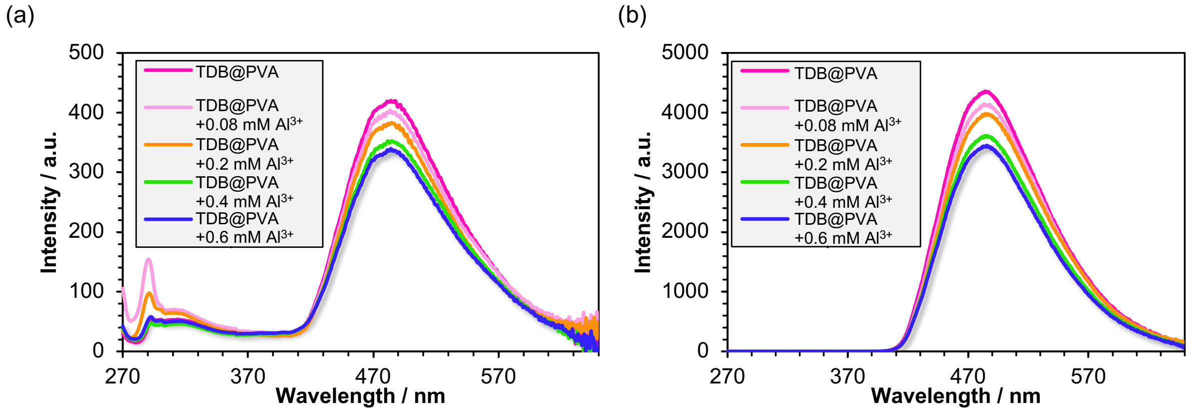

Figure S9. PL spectra(a) and time-gated PL spectra (b) of **TDB@PVA** adding several concentrations of Al^{3+} (λ_{ex} = 254 nm, delay time: 50 ms, [**TDB**] = 0.5 mol%, room temperature).

13 Comparison of the emission decay profiles of 1-TDB@PVA and 1-TDB@PVA with 0.6 mM of Al^{3+}

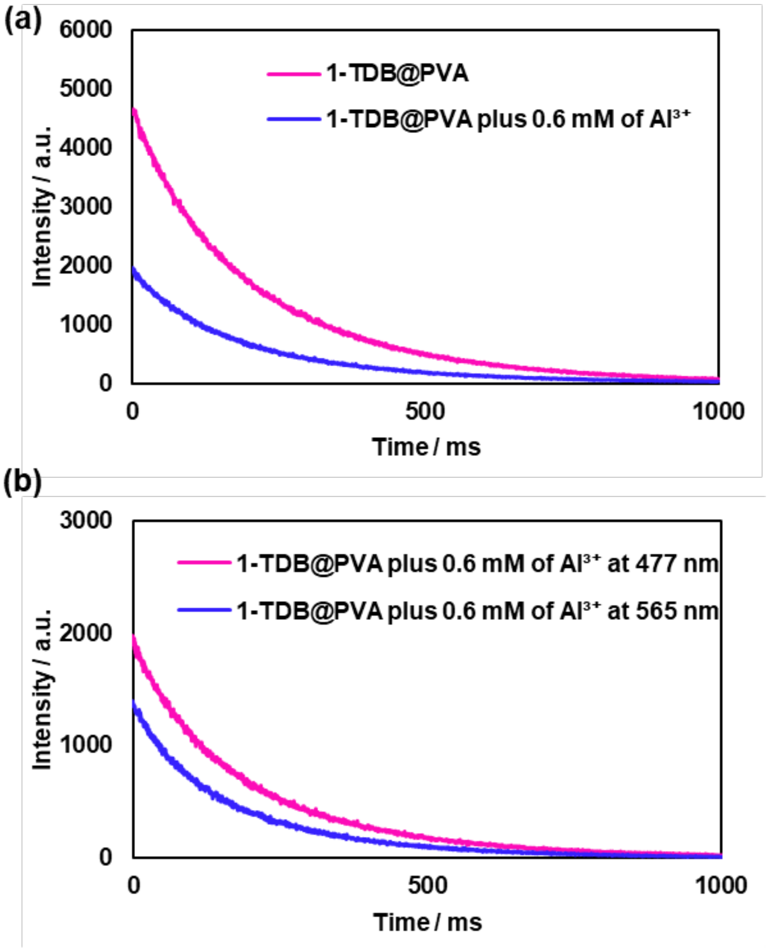

Figure S10. (a) Emission decay profiles of **1-TDB@PVA** and **1-TDB@PVA** with 0.6 mM of Al^{3+}. λ_{ex} = 254 nm, λ_{em} = 477 nm, room temperature. (b) The emission wavelength dependency.

14 Transition energies and characters of each excited states and geometry, calculated by TD-DFT (TD-ωB97X-D3/def2-TZVP)

Table S1. Transition energies and characters of **TDB** ester with ground state optimized structure.

State	Energy / eV	Wavelength / nm	Oscillator strength	Transition
S_1	5.08178	243.98	0.4753	HOMO→LUMO (95.8%)
S_2	5.22837	237.14	0.0572	HOMO–1→LUMO (91.1%)
T_1	3.16436	391.82	-	HOMO→LUMO (90.1%)
T_2	3.90315	317.65	-	HOMO–1→LUMO (94.7%)

Table S2. Transition energies and characters of **TDB** ester with S_1 optimized structure.

State	Energy / eV	Wavelength / nm	Oscillator strength	Transition
S_1	3.76470	329.33	0.3289	HOMO→LUMO (92.5%)
S_2	4.58338	270.51	0.0575	HOMO–1→LUMO (84.1%)
T_1	1.90412	651.14	-	HOMO→LUMO (95.4%)
T_2	3.30308	375.36	-	HOMO–1→LUMO (96.6%)

Table S3. Transition energies and characters of **TDB** ester with T_1 optimized structure.

State	Energy / eV	Wavelength / nm	Oscillator strength	Transition
S_1	4.66088	266.01	0.1960	HOMO→LUMO (92.3%)
S_2	5.21868	237.58	0.0328	HOMO–1→LUMO (83.9%)
T_1	2.64881	468.08	-	HOMO→LUMO (94.8%)
T_2	3.81156	325.29	-	HOMO–1→LUMO (98.3%)

Table S4. Transition energies and characters of **ATB** ester with ground state optimized structure.

State	Energy / eV	Wavelength / nm	Oscillator strength	Transition
S_1	4.29075	288.96	0.0000	HOMO–1→LUMO (69.1%)
S_2	4.81933	257.26	0.4220	HOMO→LUMO (69.2%)
T_1	2.88128	430.31	-	HOMO→LUMO (90.4%)
T_2	3.77512	328.43	-	HOMO–1→LUMO (89.4%)

Table S5. Transition energies and characters of **ATB** ester with S_1 optimized structure.

State	Energy / eV	Wavelength / nm	Oscillator strength	Transition
S_1	3.30795	374.81	0.0000	HOMO–1→LUMO (84.3%)
S_2	4.47113	277.30	0.4545	HOMO→LUMO (95.6%)
T_1	2.31558	535.44	-	HOMO→LUMO (91.3%)
T_2	2.82326	439.15	-	HOMO-1→LUMO (81.5%)

Table S6. Transition energies and characters of **ATB** ester with T_1 optimized structure.

State	Energy / eV	Wavelength / nm	Oscillator strength	Transition
S_1	3.68113	422.02	0.0000	HOMO–1→LUMO (91.5%)
S_2	4.45810	4.0161	0.5912	HOMO→LUMO (92.0%)
T_1	2.32566	533.11	-	HOMO→LUMO (95.2%)
T_2	3.21783	489.30	-	HOMO-1→LUMO (79.8%)

15 NMR spectra

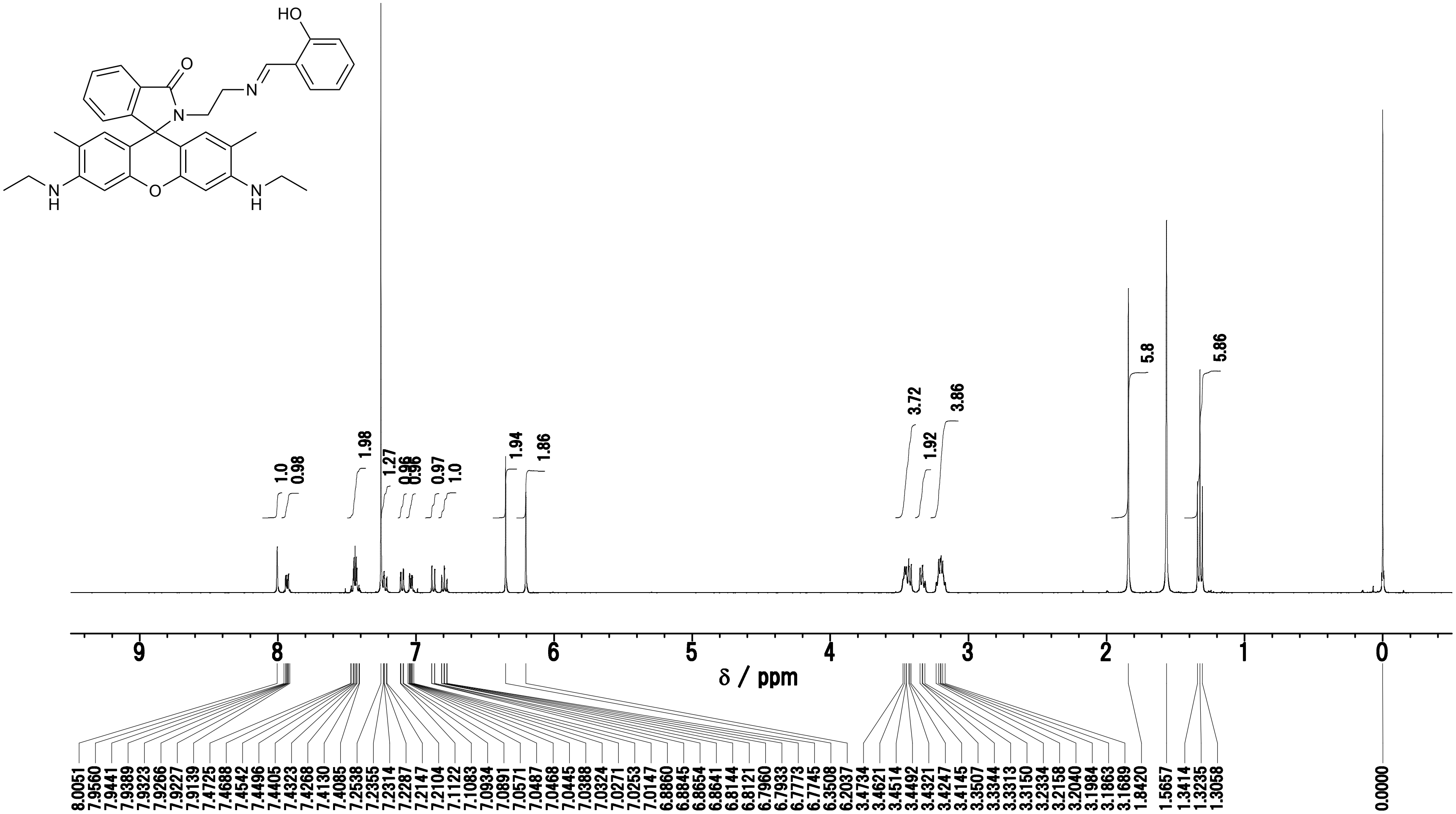

Figure S11. ^{1}H NMR spectrum (400 MHz) of **1** in $CDCl_3$.

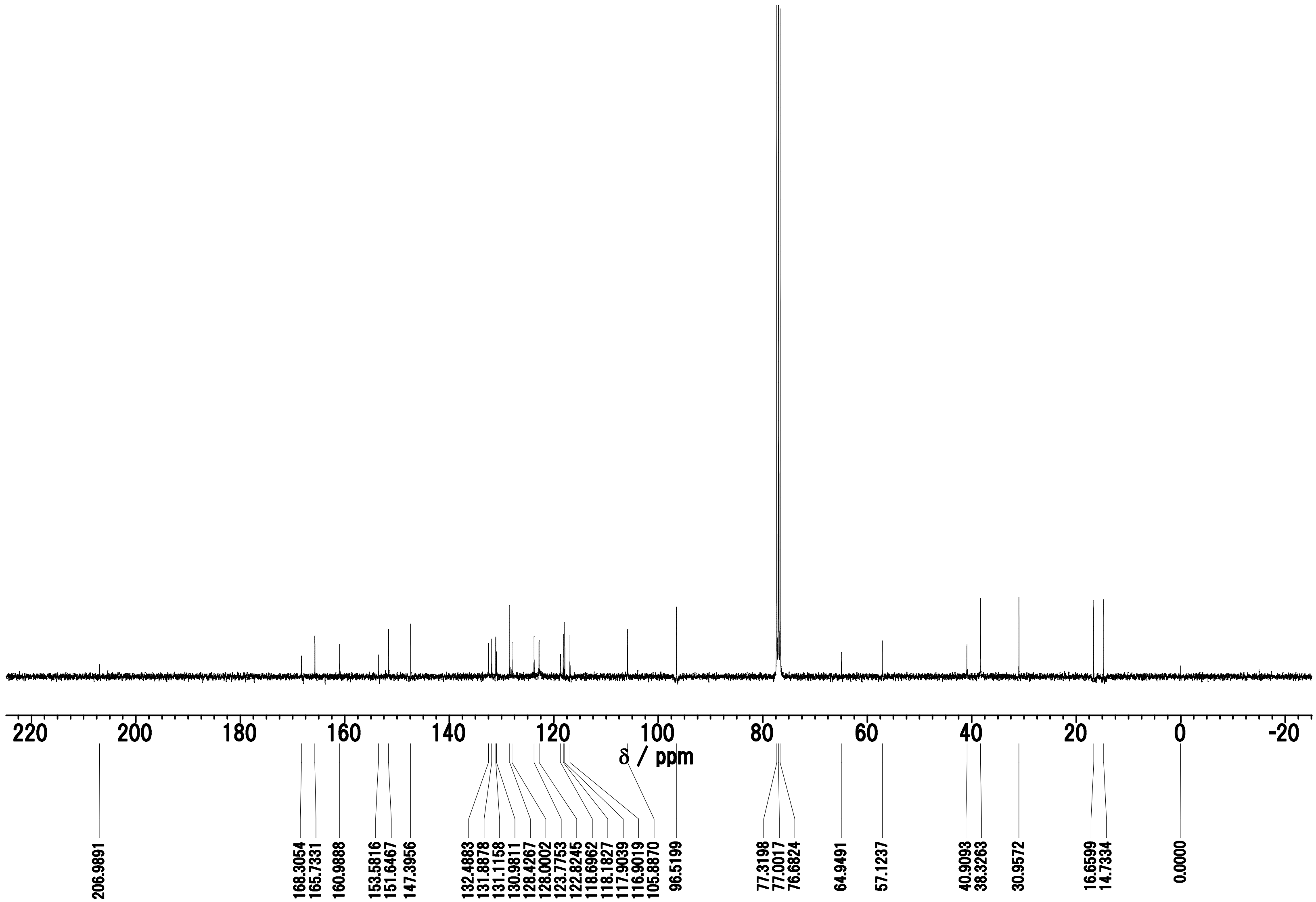

Figure S12. ^{13}C NMR spectrum (101 MHz) of **1** in $CDCl_3$.

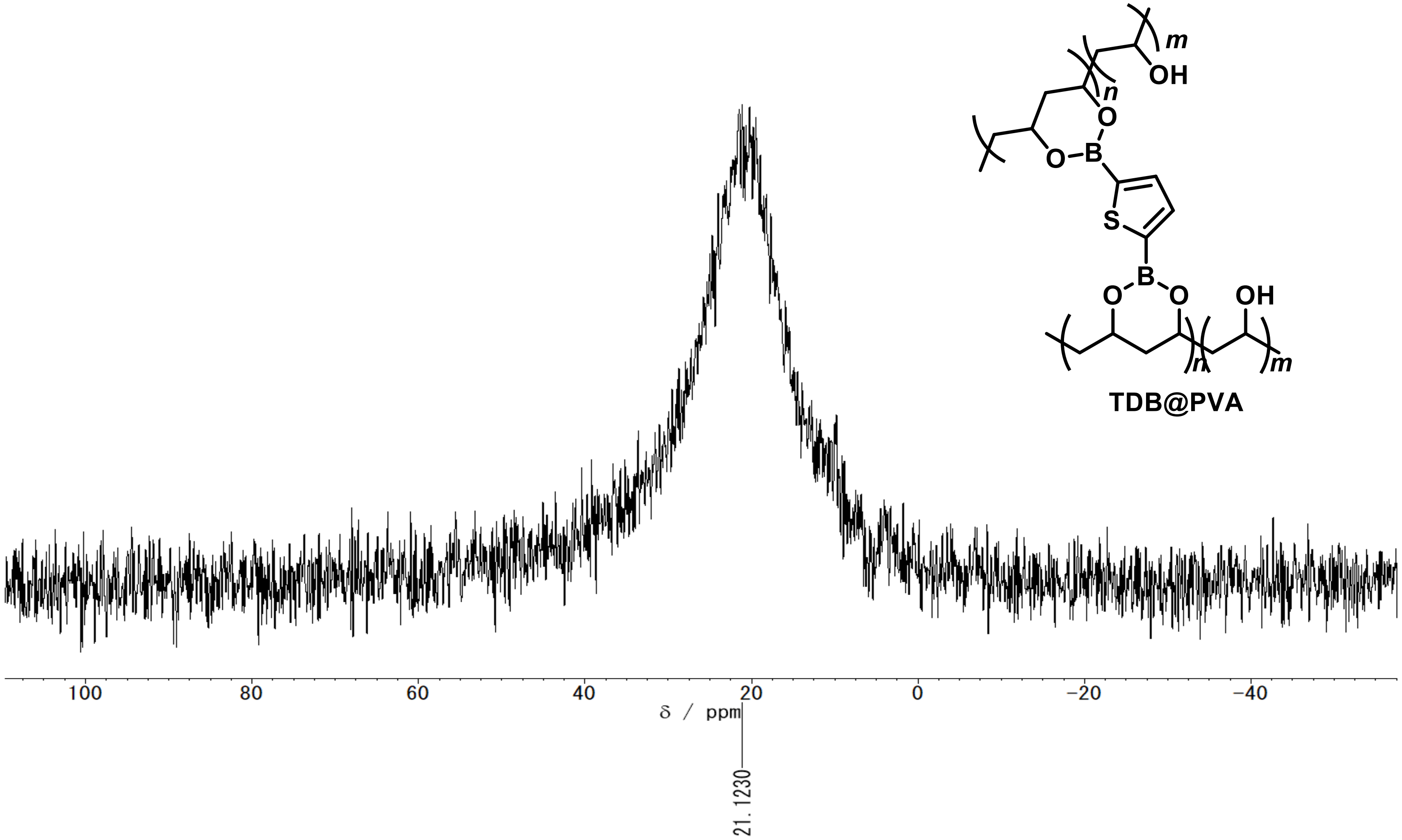

Figure S13. ^{11}B NMR spectrum (128 MHz) of **TDB@PVA** in DMSO-d_6.

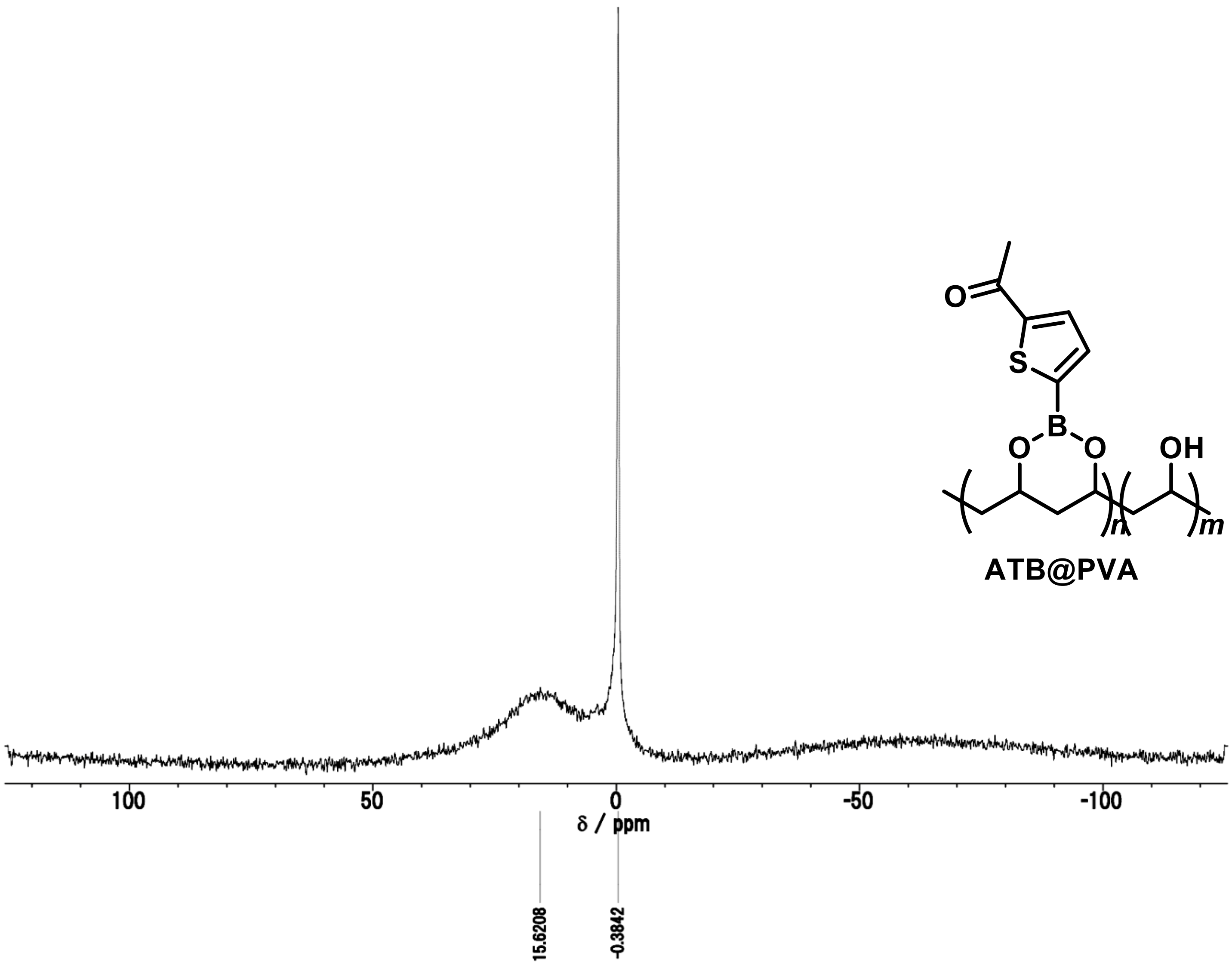

Figure S14. ^{11}B NMR spectrum (128 MHz) of **ATB@PVA** in DMSO-d_6.

16 MS spectra

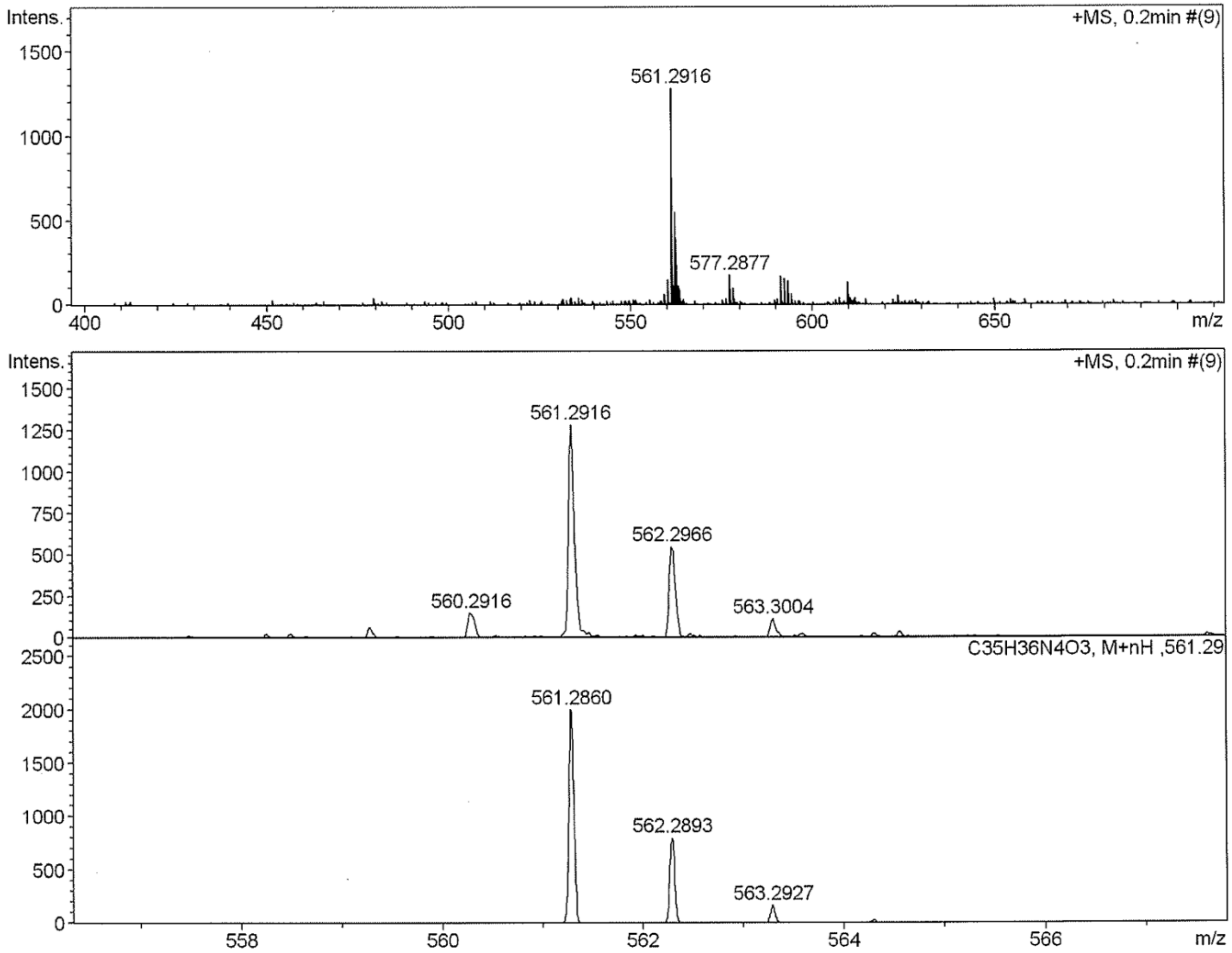

Figure S15. HR APCI-MS spectrum of **1**.

17 ATR-FTIR spectra

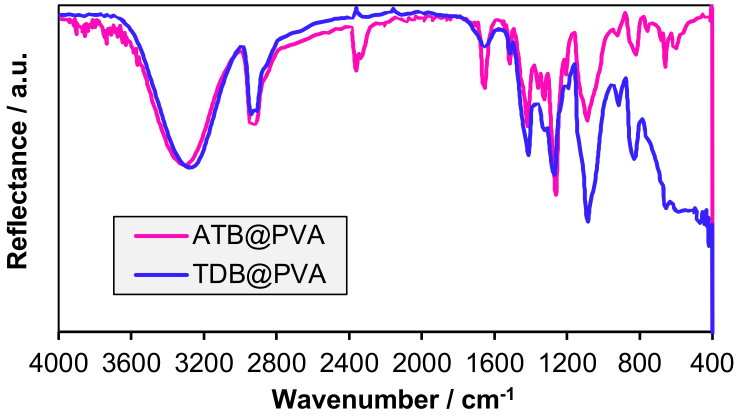

Figure S16. ATR-FTIR spectra of **TDB@PVA** and **ATB@PVA**. A characteristic peak owing to the carbonyl group appeared in **ATB@PVA**.

18 Cartesian coordinates of optimized structures (TD-ωB97X-D3/def2-TZVP)

Table S7. Cartesian coordinates of the ground state optimized structure of **TDB** ester.

	x	y	z
C	-4.79470	-1.25957	0.64061
B	0.20883	-2.53326	0.10626
O	0.20705	-2.78752	-1.24346
C	0.82503	-3.96625	-1.72947
C	0.75445	-5.08768	-0.70153
C	1.34512	-4.60612	0.61689
O	0.75430	-3.38826	1.03787
C	-0.32372	-0.70779	1.95941
C	-0.47368	0.65704	1.96625
C	-0.74729	1.17485	0.65265
B	-0.35782	2.57494	0.13343
O	-0.41489	2.83582	-1.21343
C	-0.06885	4.12104	-1.69919
C	0.89427	4.83293	-0.75829
C	0.32072	4.83070	0.65239
O	0.00612	3.51390	1.07308
S	-1.28505	-0.11044	-0.41223
H	0.31509	-4.24480	-2.65678
H	1.87263	-3.74530	-1.97992
H	-0.29182	-5.37857	-0.55162
H	1.29759	-5.96873	-1.05992
H	1.19000	-5.34130	1.41302

H	2.42907	-4.45411	0.51253
H	0.01525	-1.31480	2.79092
H	-0.27507	1.31488	2.80445
H	0.37212	3.98920	-2.69225
H	-0.98742	4.71278	-1.81988
H	1.86015	4.31426	-0.75925
H	1.06680	5.85979	-1.09824
H	1.03561	5.24213	1.37193
H	-0.58716	5.44944	0.69504

Table S8. Cartesian coordinates of the S_1 optimized structure of **TDB** ester.

	x	y	z
C	-0.47947	-1.25957	0.64061
B	0.20883	-2.53326	0.10626
O	0.20705	-2.78752	-1.24346
C	0.82503	-3.96625	-1.72947
C	0.75445	-5.08768	-0.70153
C	1.34512	-4.60612	0.61689
O	0.75430	-3.38826	1.03787
C	-0.32372	-0.70779	1.95941
C	-0.47368	0.65704	1.96625
C	-0.74729	1.17485	0.65265
B	-0.35782	2.57494	0.13343
O	-0.41489	2.83582	-1.21343
C	-0.06885	4.12104	-1.69919
C	0.89427	4.83293	-0.75829
C	0.32072	4.83070	0.65239
O	0.00612	3.51390	1.07308
S	-1.28505	-0.11044	-0.41223
H	0.31509	-4.24480	-2.65678
H	1.87263	-3.74530	-1.97992
H	-0.29182	-5.37857	-0.55162
H	1.29759	-5.96873	-1.05992
H	1.19000	-5.34130	1.41302
H	2.42907	-4.45411	0.51253
H	0.01525	-1.31480	2.79092
H	-0.27507	1.31488	2.80445
H	0.37212	3.98920	-2.69225
H	-0.98742	4.71278	-1.81988
H	1.86015	4.31426	-0.75925
H	1.06680	5.85979	-1.09824
H	1.03561	5.24213	1.37193
H	-0.58716	5.44944	0.69504

Table S9. Cartesian coordinates of the T_1 optimized structure of **TDB** ester.

	x	y	z
C	0.14459	-1.25920	0.48979
B	0.30892	-2.74181	0.12023
O	0.34003	-3.07752	-1.20988
C	0.48578	-4.43491	-1.59204
C	-0.07588	-5.36853	-0.52843
C	0.55035	-5.03912	0.82019
O	0.40804	-3.66292	1.13492
C	0.07777	-0.67111	1.82817
C	-0.07497	0.67043	1.82873
C	-0.14188	1.25962	0.49092
B	-0.31143	2.74190	0.12245
O	-0.34851	3.07752	-1.20736
C	-0.47930	4.43527	-1.59354
C	0.07574	5.36806	-0.52560
C	-0.55827	5.03743	0.81900
O	-0.40617	3.66314	1.13767
S	0.00251	0.00083	-0.74639
H	-0.03202	-4.56392	-2.54728
H	1.55129	-4.64444	-1.76004
H	-1.16310	-5.24126	-0.46835
H	0.12321	-6.41213	-0.79427
H	0.07736	-5.60993	1.62521
H	1.61941	-5.29326	0.81567
H	0.14929	-1.29588	2.71134
H	-0.14571	1.29455	2.71242
H	0.05145	4.55875	-2.54247
H	-1.54111	4.65140	-1.77547
H	1.16257	5.24109	-0.45800
H	-0.12169	6.41175	-0.79226
H	-0.09647	5.61399	1.62634
H	-1.62936	5.28281	0.80511

Table S10. Cartesian coordinates of the ground state optimized structure of **ATB** ester.

	x	y	z
C	0.01278	0.26882	-2.26911
C	0.03857	-0.13646	-3.69253
O	0.06599	0.70439	-4.56203
C	0.03023	-1.60521	-4.04919
C	0.02053	1.56541	-1.82104
C	-0.01015	1.64952	-0.41766
C	-0.04106	0.42270	0.20276
B	-0.08318	0.13502	1.73283

O	-0.11049	-1.16176	2.15572
C	-0.13944	-1.45809	3.54534
C	0.50628	-0.34811	4.35382
C	-0.12073	0.98472	3.98886
O	-0.08746	1.20005	2.58460
S	-0.03120	-0.82774	-0.95560
H	-0.52381	-2.14025	-3.84685
H	0.42462	-2.3314	-3.84642
H	0.09038	-1.39530	-4.93962
H	0.04777	2.40503	-2.50126
H	-0.01087	2.57699	0.13906
H	0.38307	-2.40563	3.68246
H	-1.18196	-1.60165	3.84790
H	1.57894	-0.31818	4.14236
H	0.38395	-0.54135	5.42163
H	0.41138	1.81512	4.45467
H	-1.16343	1.02541	4.32144

Table S11. Cartesian coordinates of the S_1 optimized structure of **ATB** ester.

	x	y	z
C	0.00738	0.26255	-2.27867
C	0.04562	-0.14742	-3.62109
O	0.07234	0.72949	-4.56932
C	0.05771	-1.56978	-4.11421
C	0.00036	1.57674	-1.81241
C	-0.03484	1.63551	-0.41170
C	-0.05728	0.41091	0.20900
B	-0.09262	0.13212	1.72877
O	-0.13557	-1.16414	2.16523
C	-0.16361	-1.44360	3.55596
C	0.52803	-0.34734	4.34389
C	-0.07919	0.99623	3.98408
O	-0.07295	1.20269	2.58089
S	-0.03580	-0.85676	-0.95783
H	-0.81706	-1.76353	-4.73908
H	0.04686	-2.24395	-3.25988
H	0.95311	-1.75738	-4.71144
H	0.02194	2.43582	-2.46699
H	-0.04385	2.55765	0.15467
H	0.32402	-2.40828	3.70332
H	-1.20763	-1.54385	3.87322
H	1.59506	-0.34354	4.10338
H	0.42888	-0.53109	5.41585
H	0.48070	1.81671	4.43572
H	-1.11268	1.05853	4.34267

Table S12. Cartesian coordinates of the T_1 optimized structure of **ATB** ester.

	x	y	z
C	-2.28803	0.24551	0.15183
C	-3.67614	-0.10514	0.23975
O	-4.51883	0.79501	0.37569
C	-4.06803	-1.56385	0.16383
C	-1.78373	1.62485	0.21376
C	-0.43571	1.69207	0.10986
C	0.21663	0.41610	-0.03755
B	1.72987	0.13708	-0.17761
O	2.13274	-1.16363	-0.30537
C	3.51680	-1.46537	-0.41790
C	4.36791	-0.39769	0.25608
C	3.98517	0.97639	-0.27726
O	2.58678	1.20402	-0.15808
S	-0.96803	-0.90497	-0.04114
H	-3.73883	-2.00996	-0.78123
H	-3.60461	-2.13656	0.97488
H	-5.15241	-1.64365	0.24157
H	-2.47102	2.45225	0.33126
H	0.14460	2.60776	0.13126
H	3.67440	-2.44653	0.03895
H	3.77282	-1.54670	-1.48261
H	4.20350	-0.42826	1.33948
H	5.43049	-0.59107	0.07593
H	4.49277	1.77194	0.27569
H	4.26812	1.07038	-1.33424
